# PKC-η-MARCKS Signaling Promotes Intracellular Survival of Unopsonized *Burkholderia thailandensis*

**DOI:** 10.3389/fcimb.2017.00231

**Published:** 2017-06-07

**Authors:** Sofiya N. Micheva-Viteva, Yulin Shou, Kumkum Ganguly, Terry H. Wu, Elizabeth Hong-Geller

**Affiliations:** ^1^Bioscience Division, Los Alamos National LaboratoryLos Alamos, NM, United States; ^2^Center for Infectious Disease and Immunity and Department of Internal Medicine, University of New Mexico Health Sciences CenterAlbuquerque, NM, United States

**Keywords:** *Burkholderia*, infection, RNA interference, autophagy, intracellular bacteria, protein kinase C

## Abstract

Pathogenic *Burkholderia* rely on host factors for efficient intracellular replication and are highly refractory to antibiotic treatment. To identify host genes that are required by *Burkholderia* spp. during infection, we performed a RNA interference (RNAi) screen of the human kinome and identified 35 host kinases that facilitated *Burkholderia thailandensis* intracellular survival in human monocytic THP-1 cells. We validated a selection of host kinases using imaging flow cytometry to assess efficiency of *B. thailandensis* survival in the host upon siRNA-mediated knockdown. We focused on the role of the novel protein kinase C isoform, PKC-η, in *Burkholderia* infection and characterized PKC-η/MARCKS signaling as a key event that promotes the survival of unopsonized *B. thailandensis* CDC2721121 within host cells. While infection of lung epithelial cells with unopsonized Gram-negative bacteria stimulated phosphorylation of Ser175/160 in the MARCKS effector domain, siRNA-mediated knockdown of PKC-η expression reduced the levels of phosphorylated MARCKS by >3-fold in response to infection with Bt CDC2721121. We compared the effect of the conventional PKC-α and novel PKC-η isoforms on the growth of *B. thailandensis* CDC2721121 within monocytic THP-1 cells and found that ≥75% knock-down of PRKCH transcript levels reduced intracellular bacterial load 100% more efficiently when compared to growth in cells siRNA-depleted of the classical PKC-α, suggesting that the PKC-η isoform can specifically mediate *Burkholderia* intracellular survival. Based on imaging studies of intracellular *B. thailandensis*, we found that PKC-η function stimulates phagocytic pathways that promote *B. thailandensis* escape into the cytoplasm leading to activation of autophagosome flux. Identification of host kinases that are targeted by *Burkholderia* during infection provides valuable molecular insights in understanding *Burkholderia* pathogenesis, and ultimately, in designing effective host-targeted therapies against infectious disease caused by intracellular pathogens.

## Introduction

Intracellular bacteria often defy the traditional pathogen-directed antimicrobial therapies through adaptation mechanisms that co-opt host cell processes in order to maximize infection efficiency. Pathogenic *Burkholderia* species, including *B. pseudomallei* and *B. mallei*, the causative agents of human melioidosis and equine glanders, respectively, are intracellular pathogens of particular concern, since infections are highly refractory to a broad spectrum of antibiotics and are associated with a mortality rate of ~50% even with antibiotic treatment (Moore et al., 1999; Wiersinga et al., 2006; Currie et al., 2010; Schweizer, 2012). *Burkholderia* spp. employ two types of secretion systems, Type III (T3SS) and Type VI (T6SS), consisting of multiple effector proteins that mediate host invasion and intracellular survival, thus enabling *Burkholderia* avoidance of the host immune responses, resistance to antibiotic treatment, and establishment of latent infections (Stevens et al., 2003; Burtnick et al., 2008, 2009, 2011; Muangsombut et al., 2008; Gong et al., 2011). Chronic infections caused by *B. pseudomallei* are a major challenge to achieving sterile immunity and a contributing factor to disease spread outside of the endemic zone, Southeast Asia and northern Australia (Limmathurotsakul et al., 2016). Sporadic infections with *B. pseudomallei* have been recorded in East Africa, the Caribbean, Central and South America, the Middle East, North America and Western Europe, emphasizing the effect of globalization on emerging infectious diseases (Doker et al., 2014; Benoit et al., 2015; Currie, 2015).

In pursuit of effective treatment of persistent *Burkholderia* infections, several host transcriptomics studies have been performed to characterize changes in host gene expression in response to *Burkholderia* infection (Ulett et al., 2005; Chin et al., 2010; Mariappan et al., 2013). Host genes that function in apoptosis, immune response, stress response, and cellular metabolism were found to be differentially regulated upon infection. Yeast two-hybrid screens of whole human and murine proteome libraries have identified 600 human and 846 murine protein interactions with *B. mallei* virulence factors, demonstrating high representation of host proteins that function in ubiquitination, phagosome formation, and actin cytoskeleton dynamics (Memisevic et al., 2013, 2015). To further characterize host gene function in response to *Burkholderia* infection, we performed a RNA interference (RNAi) screen of the human kinome to identify host factors that facilitate intracellular survival of *Burkholderia thailandensis*, a surrogate for the more virulent *B. pseudomallei*. Although ~1,000-fold less lethal than *B. pseudomallei* in animal models, *B. thailandensis* nevertheless induces phagocytic mechanisms and exhibits growth kinetics in primary human monocyte-derived dendritic cells similar to *B. pseudomallei* (Wiersinga et al., 2006; Haraga et al., 2008; Charoensap et al., 2009). To validate the results of our RNAi screen, we used the clinical isolate *B. thailandensis* CDC2721121 previously shown to exhibit phenotypes that resemble pathogenic *Burkholderia* in cell culture studies (Glass et al., 2006). *Bt* CDC2721121 has acquired the capsular polysaccharide virulence cluster, and thus exhibits *B. pseudomallei*-like phenotypes, including colony wrinkling, resistance to human complement binding, and survival within macrophages (Sim et al., 2010).

Our RNAi screen revealed divergent roles for protein kinase C (PKC) isoform functions in promoting intracellular proliferation of *B. thailandensis*. The PKC family comprises at least 10 related lipid-sensitive serine/threonine kinases subdivided into three classes with particular features: (1) the classic PKCs (PKCα, PKCβI, PKCβII, PKCγ), which are Ca^2+^-dependent and activated by both PS and DAG; (2) the novel PKCs (PKCδ, PKCε, PKCη, PKCμ, PKCθ), which require DAG but not Ca^2+^; and (3) atypical PKCs (PKCζ, PKCί/λ), which are Ca^2+^- and DAG-independent (Mochly-Rosen et al., 2012). Classical Ca^2+^-dependent PKC isoforms are known to be key regulators of host innate immunity and phagocytosis of opsonized antigen through the IgG receptors (Breton and Descoteaux, 2000; Underhill and Ozinsky, 2002). In our assay we have used unopsonized *B. thailandensis* as a model of early infection event to study the effect of cellular innate immune responses in the restriction of intracellular bacterial growth. We identified the novel PKC-eta isoform, PKCη, as a host factor required for the successful growth of unopsonized *B. thailandensis* within professional phagocytes and epithelial cells. Other RNAi-based screens have previously discovered various novel PKC isoforms to be required for the colonization of epithelial tissue cells by intracellular pathogenic bacteria (Prudencio et al., 2008; Jiwani et al., 2012). Similarly, these discovery platforms were infected with unopsonized bacteria. We further characterized PKC-η/MARCKS signaling as a key event that promotes uptake of unopsonized *B. thailandensis* by host cells and demonstrated that opsonization is a key factor that determines receptor usage, triggers differential PKC signaling pathways, and eventually determines intracellular pathogen survival.

## Materials and methods

### Bacterial strains and growth conditions

The following bacterial strains were used in this study: (1) *B. thailandensis* DW503, a derivative from the environmental *B. thailandensis* E264 (gift from Dr. Mary Burtnick, University of South Alabama) (Burtnick et al., 2001); (2) *B. thailandensis* CDC2721121, a clinical isolate obtained from the CDC (Glass et al., 2006); and (3) *Yersinia enterocolitica* WA (pYV^+^, ATCC 27729). Both *Burkholderia* strains were grown on LB agar plates at 37°C and stored at 4°C for up to a week or were cultured on LB broth with aeration at 37°C prior to the infection of host cells. The *B. thailandensis* DW503-GFP strain was generated by introduction of the BHR4-GFP plasmid (gift from Dr. M. Burtnick) into bacterial cells via electroporation and selection of clones using LB agar plates containing 50 μg/ml gentamicin. For cell infection experiments, *Y. enterocolitica* WA was grown at 26°C in brain heart infusion (BHI, Difco, Detroit, Mich) broth for 18 h in an orbital shaker at 180 rpm, followed by dilution of the bacterial culture to obtain 0.1 OD_660_ and additional growth for 2 h at 37°C (100 rpm) to stimulate production of the T3SS.

### Tissue culture cell growth conditions

THP-1 human monocytes (ATCC TIB-202) were maintained in RPMI-1640 GlutaMax media (Invitrogen, Carlsbad, CA) supplemented with 10% FBS (HyClone, Logan, UT). A549 cells (ATCC CCL-185) were cultured in DMEM high glucose medium containing 10% FBS (Invitrogen) at 37°C and 5% CO_2_. The RFP-LC3B stable cell line was generated by transfection of A549 cells with pmRFP-LC3 (Addgene plasmid #21075) (Kimura et al., 2007) and flow cytometry sorting using the BD FACSAria. Phorbol 12-myristate 13-acetate (PMA) and mannan from *S. cerevisiae* were obtained from Sigma-Aldrich (St. Louis, MO).

### Validation screen using imaging flow cytometry

We validated host gene function in intracellular bacterial replication using a differential two-step labeling method to distinguish between intracellular and extracellular *B. thailandensis*. *Bt* CDC2721121 cells (10^8^) were incubated in 1 ml PBS (pH6) containing 1 μg/ml FITC (green) for 15 min at room temperature, washed four times in 1 ml PBS, and resuspended in LB. THP-1 cells were treated with siRNAs as described for the screen and infected with FITC-labeled *Bt* CDC2721121 at MOI 10, 72 h post-siRNA treatment. Growth of extracellular bacteria was suppressed by adding kanamycin (250 μg/ml) 2 h post-infection. After another 3 h, the infected THP-1 cells were pelleted at 800 g for 2 min and resuspended in fresh antibiotic-free RPMI/10% FBS. THP-1 cells were collected 24 h post-infection, fixed for 20 min in 1% paraformaldehyde, and incubated for 1 h in blocking buffer containing 10% sheep serum and 1% bovine serum albumin (BSA). Rabbit anti-*B. thailandensis* serum (gift of Dr. Paul Brett, University of South Alabama) was then added (1:500 dilution) to the blocking buffer, and cells were incubated for an additional 1 h. Following three rounds of washing in PBS, THP-1 cells were incubated for 30 min in PBS containing 1% BSA and PE (red)-conjugated anti-rabbit goat IgG (1:1,000 dilution). Thus, extracellular adherent *Burkholderia* were double-labeled with FITC and PE fluorescence, whereas intracellular bacteria were single-labeled with FITC. The labeled THP-1 cells were analyzed using imaging flow cytometry (Amnis ImageStream^X^) with the following data collection parameters: slow flow rate, 60 × magnification, and extended depth of field. Images were collected from the darkfield, brightfield, FL1 (488ex/560em nm), and FL2 (488ex/595em nm) channels. Uninfected THP-1 cells treated with rabbit anti-*B. thailandensis* sera and PE-conjugated anti-rabbit IgG treatment, and *Bt* CDC2721121-FITC infected THP-1 cells not treated with PE-conjugated anti-rabbit IgG, served as single color controls to establish compensation that excluded channel cross-talk. The IDEAS 5.0 software was used to define the localization of internalized bacteria and to determine the percentage of single vs. double-labeled THP-1 cells.

### Western blot analysis

THP-1 cells treated with 50 nM siRNA were washed twice with chilled PBS and lysed on ice for 45 min with RIPA Lysis and Extraction Buffer (Thermo Scientific, Grand Island, NY) supplemented with a cocktail of protease and phosphatase inhibitor mixture (Thermo Scientific). Lysates were centrifuged (12000 rpm, 15 min), supernatants were collected, and total protein was quantified with the BCA kit (Pierce). Equal amounts of protein (10 μg) were run on SDS–PAGE and transferred to PVDF membranes (Millipore, Billerica, MA) blocked with 5% nonfat dried milk in TBST (25 mM Tris [pH 7.6], 137 mM NaCl, and 0.2% Tween 20). Membranes were incubated with primary antibodies to PKC-α (sc-8393, H-7), PKC-η (sc-215, C-15), and actin (sc-58673, 2Q1055) (Santa Cruz Biotechnology, Santa Cruz, CA) at 4°C overnight, washed with TBST, and incubated with HRP-conjugated secondary antibody. Immunoreactive proteins were visualized with luminol reagent (Santa Cruz Biotechnology).

### Cytotoxicity assays

The CytoTox-ONE™ Assay (Promega, Madison, WI) was used to measure the release of lactate dehydrogenase (LDH) from cells with a damaged membrane. Normalized LDH activity was calculated by subtracting the fluorescent readings in the supernatants of untreated control cells from the experimental sample readings. The percentage of necrotic cells was determined as the fluorescence signal measured in the experimental sample relative to the signal obtained from a control sample treated with 0.1% Triton and processed in parallel. The results were analyzed using the Synergy2 Multi-Mode Microplate Reader (BioTec, Winooski, VT) at 560 nm excitation and 590 nm emission.

### ELISA to determine levels of phosphorylated marcks protein

A549 cells (2 × 10^5^) were washed twice in ice-cold PBS and resuspended in 250 μl ice-cold Cell Lysis Buffer (Cell Signaling Technology, Danvers, MA) supplemented with 1 mM PMSF and 1X Halt Protease Inhibitor cocktail (ThermoScientific). Whole cell lysates were subjected to centrifugation at 12,000 g for 10 min at 4°C, and the resulting supernatants were analyzed using the PathScan Phospho-MARCKS (Ser152/156) Sandwich ELISA Kit (Cell Signaling Technology) following the manufacturer's instructions. Whole cell lysates (in triplicate) were incubated with pMARCKS antibody-coated microwells overnight at 4°C.

### *B. thailandensis* intracellular survival

Overnight cultures of A549 cells were infected with *B. thailandensis* CDC2721121 at the indicated MOI for 2 h at 37°C. Cells were washed with PBS and resuspended in DMEM containing 250 μg/ml kanamycin to suppress the growth of extracellular bacteria. After 3 h of antibiotic exposure, cells were washed 3 × with PBS and further cultured in antibiotic-free DMEM. Prior to analysis, cells were washed with warm PBS and lysed in 0.1% Triton X-100 in PBS for 5 min. The lysis mixture was diluted, plated on LB agar plates, incubated overnight at 37°C for growth, and then quantified by colony counts. The replication index was calculated as the fold increase of intracellular bacteria between two time points.

### Analysis of multinucleated giant cell (MNGC) formation

A549 cells infected with *Bt* CDC2721121 in 6-well plates were washed with PBS and treated with acid ethanol [5% acetic acid (v/v), 5% dH2O and 90% ethanol (v/v)] for 30 min at room temperature. Cells were washed twice with PBS and stained with Giemsa solution (0.1% w/v) for 30 s at room temperature. After washing with dH2O, cells were air-dried and analyzed using a light microscope. At least 10 fields per view at 10 × magnification were analyzed to obtain the percentage of MNGCs, where a cell was considered a MNGC if cell fusions with 3 or more nuclei were present.

### Gene expression

Total RNA from *Burkholderia* was isolated using the RNeasy Miniprep Kit (Qiagen, Germantown, MD) and subjected to the TURBO DNA-free Kit (Life Technologies, Grand Island, NY) for DNase treatment and removal. 100 ng purified RNA was used for quantitative real-time PCR with the TaqMan RNA-to-C_*T*_ 1-Step kit following the manufacturer's instructions (Life Technologies). Transcripts were normalized to 18S ribosomal RNA levels, using the VIC-MGB primer limited set, and calculated relative to the control sample using the $2_{\text{T}}^{-\Delta \Delta \text{C}}$ method.

### Imaging flow cytometry to quantify autophagosomes and cytoplasmic *B. thailandensis*

A549 cells expressing RFP-LC3B were grown on Corning-Costar 96-well plates with coverslip bottoms and treated with 10 μM rapamycin or infected with *Bt* CDC2721121 at MOI 10. Cells were fixed with 4% paraformaldehyde 5 h post-treatment, washed with PBS, and stored in ProLong Gold mount with DAPI nuclear stain (LifeTechnologies). Samples were acquired at optimized laser strength (100 mW for 488) with an area classifier (number of pixels in μm2) set at 50 using a Zeiss inverted fluorescence microscope. Images were acquired for each cell at 60 × magnification and enhanced depth of perception. ~2,000 cells were analyzed for each experimental or isotype control sample, and 500 cells were acquired for each single positive compensation control sample. The integrated software INSPIRE (version 6.0.154, Amnis) was used for data collection. Gating was determined for each experimental sample and cut-offs were defined using an untreated A549-RFP-LC3 control sample. All single and focused cells used in our analysis had zero saturated pixels. LC3 positive spots were identified by creating a spot mask for peak intensities that were greater than background. The peak to background ratio was determined for each experiment based on untreated controls. This spot mask was then converted into a spot count feature. The bacteria and LC3 spot counts were plotted against a normalized frequency of cells to generate histograms. To determine the bacterial counts in the cytoplasm that were not associated with LC3-positive spots, we used the following algorithm: Spot {Intensity (MO2, Ch02, 114-4095), Ch02 Bright, 16.25, 1}, and Not Spot {Intensity (M04,ch04, 240-4095), Ch04, Bright, 4, 1}. Analysis was performed on the compensated image files using algorithms in IDEAS (version 5.0, Amnis) image analysis software.

### Statistical analysis

One-way ANOVA and *Z*-test were used to evaluate the significance of responses in the HTS experiments. For samples *n* ≥ 3, the statistical significance of the difference between experimental and control data sets was determined via Student's *t*-test and the normal distributions for multiple-comparisons of experimental samples were confirmed using the Tukey-Kramer test where we specified that the entire set of comparisons should have a family error rate of 0.05.

## Results

### RNAi-based gene silencing screen of host kinases in host-*B. thailandensis* interactions

We performed a functional genomic screen using a siRNA library against 718 annotated human kinases or kinase-interacting proteins to identify host proteins required for *B. thailandensis* survival in human monocytic THP-1 cells (Supplementary Materials and Methods and Supplementary Table 1). We identified 35 initial hits (Table 1), in which siRNA-mediated gene silencing resulted in ≥50% reduction in host cell-associated bacteria. Genes encoding kinases that play a role in host cell survival pathways were highly represented in our screen. Screen hits also included kinases that regulate cell adhesion and actin dynamics, cell processes that are essential for phagocytosis of intracellular bacteria (Kuijl et al., 2007).

**Table 1:** Host genes required for intracellular *B. thailandensis* proliferation.

**Gene name**	**Accession no**.	**Biochemical activity**	**Biological function**
ABL1	NM_005157	Protein tyrosine kinase	Cell division differentiation, adhesion, and stress
AKT1/PKB	NM_005163	Serine/threonine-protein kinase	Cell survival, growth, proliferation, motility
AKT2/PKBB	NM_005163	Serine/threonine-protein kinase	Cell survival, insulin signaling, motility
AURKA	NM_198437	Cell cycle-regulated serine/threonine kinase	Organizing microtubules during cell division
AURKC	NM_198437	Serine/threonine/tyrosine kinase	Mitosis and meiosis
CALM1	NM_006888	Calcium binding phosphorylase kinase	Centrosome cycle, cytokinesis, cell signaling
CAMK1G	NM_020439	Ca^++^/calmodulin-dependent kinase	Ca^++^-triggered signaling cascade
CAMK2A	NM_015981	Serine/threonine kinase	Gene transcription, cell survival, apoptosis, cytoskeleton
CAMK2B	NM_172084	Serine/threonine kinase	Same as CAMK2A
CAMKK2	NM_172215	Ca^++^/calmodulin-dependent serine/threonine kinase	Gene transcription, cell survival, cytoskeleton reorganization
EPHA3	NM_182644	Receptor protein-tyrosine kinase	Cell signaling, development, migration
EPHB2	NM_004443	Receptor protein-tyrosine kinase	Cell signaling, development, migration, and adhesion
ERBB3/ cErbB3	NM_001005915	Receptor tyrosine kinase	Gene expression, cytoskeletal rearrangement, cell survival
HK1	NM_000188	Produce glucose-6-phosphate	Glycolysis, energy generation
HK2	NM_000189	Produce glucose-6-phosphate	Glycolysis, energy generation
HK3	NM_002115	Produce glucose-6-phosphate	Glycolysis, energy generation
INSRR	NM_014215	Transmembrane receptor protein tyrosine kinase	Cell signaling, apoptosis, pH sensor
ITPKA/IP3KA	NM_002220	Phosphorylation of inositol 1,4,5-trisphosphate to Ins (1,3,4,5) P4	Cell signaling, motility
KDR	NM_002253	Receptor protein-tyrosine kinase	Proliferation, survival, migration
MAST3	NM_015016	Serine/threonine kinase	Mitosis
PAK3	NM_002578	Serine/threonine p21-activating kinase	Cell cycle, migration
PIK3R1	NM_181523	Phosphorylation of the inositol ring of PI at the 3′ position	Cell signaling, vesicular trafficking, migration, insulin metabolism
PIK3R5/P101-PI3K	NM_14308	Phosphorylation of the 3'OH of the inositol ring of phosphoinositide (PI)	Cell growth, proliferation, differentiation, motility, and intracellular trafficking
PIP5KIA	NM_003557	Phosphorylation of 4-phosphate of PI	Endocytosis and cell migration
PIP5K2B	NM_003559	Phosphorylation of 5′-phosphate of PI at the 4′OH	Cell signaling, proliferation, motility
PKN3	NM_013355	Serine/threonine-protein kinase	Cell growth, migration, adherence
PRKCH	NM_006255	Serine/threonine-protein kinase	Cell signaling, proliferation, survival, motility
PTK7	NM_152881	Transduction of extracellular signals across the cell membrane	Wnt cell signaling, polarity, adhesion
PRKACB/PKA C-β	NM_002731	cAMP-dependent catalytic subunit of serine/threonine-protein kinase	Regulation of lipid and glucose metabolism, cell signaling
PCK1	NM_002591	Catalyzes the formation of phosphoenolpyruvate from oxaloacetate	Regulation of gluconeogenesis
STK11/LKB1	NM_000455	Serine/threonine kinase, phosphorylates AMPK	Cell polarity and survival, metabolic reprogramming
STK35/CLIK1	NM_080836	Serine/threonine kinase	Cell cycle, migration
STK38L/NDR1	NM_015000	Mg^++^ binding serine/threonine kinase	Cytoskeletal reorganization, cell invasion
STK4	NM_006282	Serine/threonine kinase	apoptosis
TYRO3	NM_006293	Receptor protein-tyrosine kinase	Cell survival, proliferation and regulation

**Figure 1:**
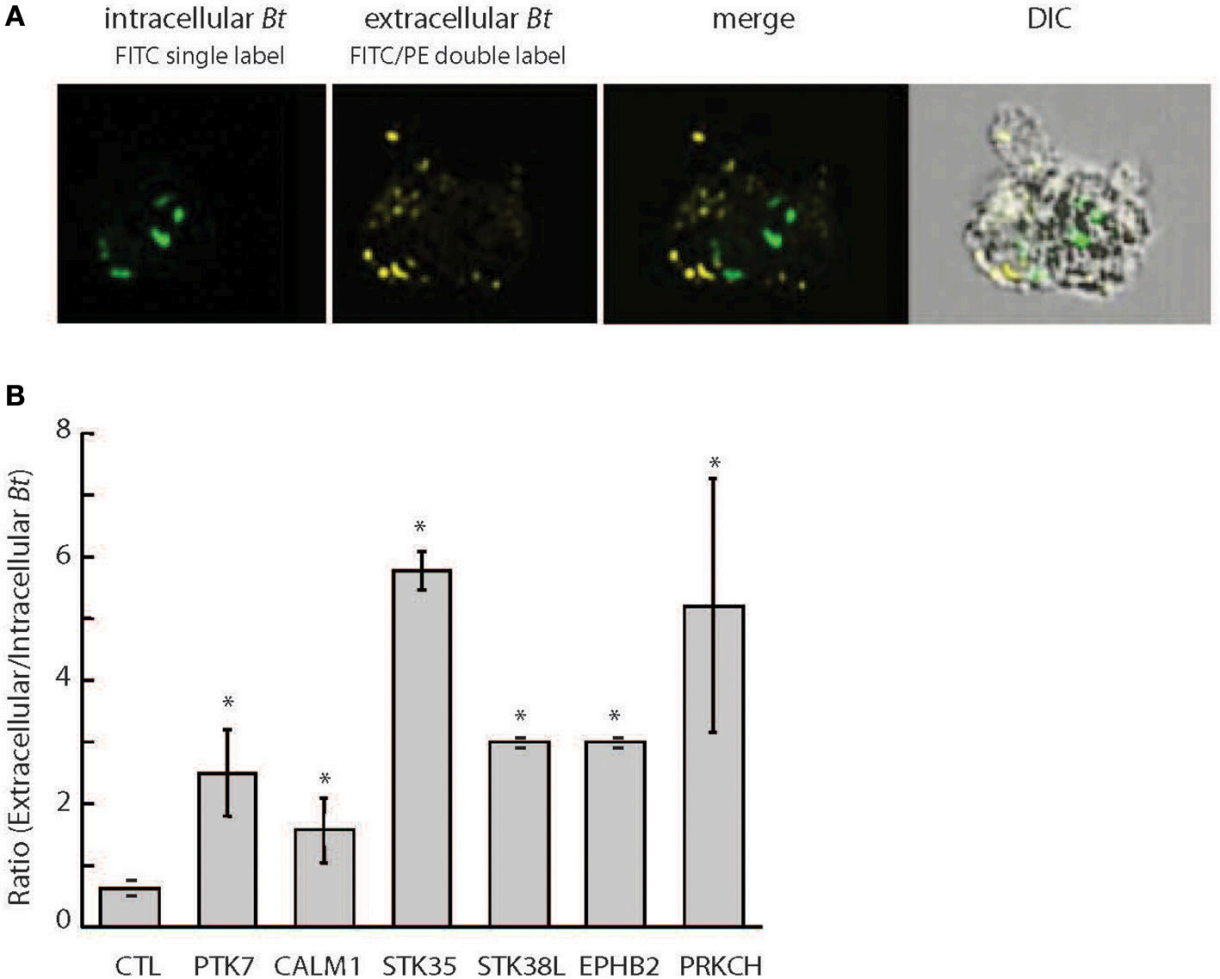
Differential fluorescent labeling of intracellular *B. thailandensis* upon siRNA knockdown of selected host kinase genes. **(A)** THP-1 cells pretreated with 50 nM siRNA for 72 h were infected with FITC-labeled *Bt* CDC2721121 for 2 h at MOI 10. Kanamycin (250 μg/ml) was then added for 3 h to suppress extracellular bacterial growth, after which the infected cells were cultured in antibiotic-free media for 20 h. Bacterial cells remaining extracellular were double-labeled with FITC and *B. thailandensis* rabbit antiserum/PE-conjugated anti-rabbit IgG, whereas intracellular bacteria were single-labeled with only FITC. Imaging flow cytometry analysis of infected THP-1 cells was performed using the Amnis ImageStream^X^, and the frequencies of singly and double labeled bacteria were determined using the IDEAS 5.0 software. FITC, fluorescein isothiocyanate (ex495 nm/em519 nm); PE, Phycoerythrin (ex 488 nm/em575 nm). **(B)** The ratio of extracellular vs. intracellular bacteria in THP-1 cells siRNA-depleted of target kinases was used to evaluate kinase function in intracellular *B. thailandensis* proliferation. The average and standard deviation were determined from three independent experiments (Supplementary Table 2). The “^*^” denotes statistical significance (*p* < 0.05) for the ratio of extracellular to intracellular pathogen in cells siRNA-depleted of target gene compared to cells treated with control siRNA.

For subsequent validation studies, we used the clinical isolate *B. thailandensis* CDC2721121. We applied differential fluorescent labeling of extracellular and intracellular *Bt* CDC2721121 to assess the efficiency of pathogen internalization and survival within THP-1 cells using imaging flow cytometry (Figure 1A). We observed that siRNA-mediated silencing of six genes regulating actin dynamics (PTK7, EPHB2, and PRKCH), calcium signaling (CALM1), cell adhesion (STK35), and cell proliferation (STK38L) resulted in a significant (z>-2) reduction of *B. thailandensis* intracellular load in THP-1 cells, compared to cells treated with control siRNA (Figure 1B, Supplementary Table 2). The RNAi silencing of target gene activity in monocytic THP-1 cells was efficient (>75%), transcript-specific (Supplementary Figure 1), and did not affect host cell viability when compared to the control cells treated with non-targeting siRNA (Supplementary Figure 2).

### Differential role of conventional and novel PKC isoforms in *B. thailandensis* survival in human monocytes

**Figure 2:**
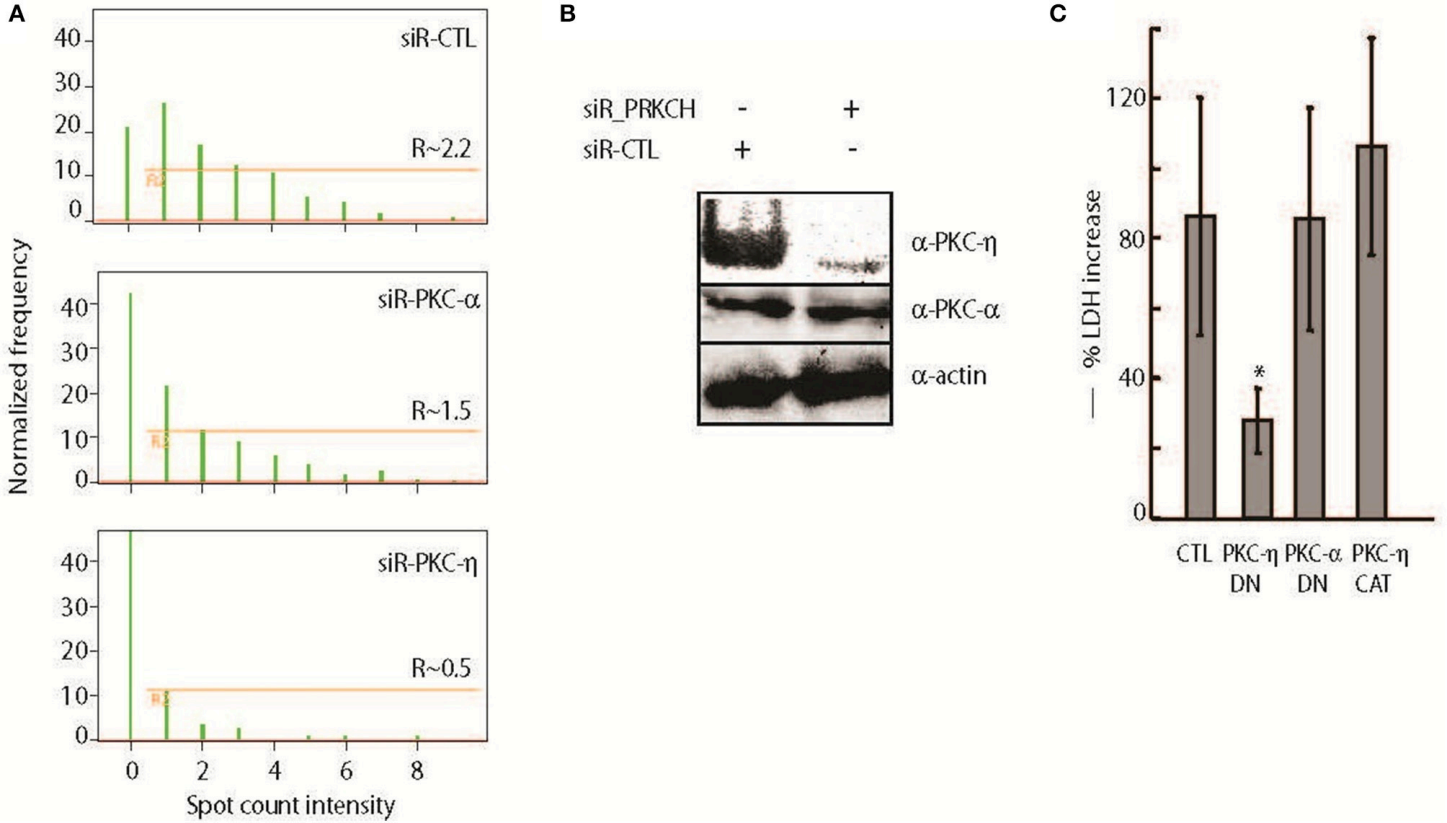
PKC-η plays bigger role than PKC-α in unopsonized *B. thailandensis* intracellular survival and induction of host cell death. **(A)** Quantitative analysis of internalized *Bt* CDC2721121 was determined by plotting bacteria spot counts, 1–2 μm fluorescent puncti corresponding to bacteria size and fluorescent marker, against a normalized frequency, percentage of analyzed cells selected from the total number of single and focused cells. Bacterial counts in the cytoplasm that were singly labeled with FITC and not associated with the PE-labeled extracellular bacterial spots were calculated as described in the Materials and Methods. R, the gate excluding the cells exhibiting background fluorescence from the analysis, shows the mean value of fluorescence (MF) spots associated with intracellular bacteria per single host cell. R and NF (Normalized frequency) were determined by selective analysis of at least 5000 single and focused cells per sample using the IDEAS 5.0 software. A representative result of one out of 3 independent experiments is shown. **(B)** THP-1 cells were transfected with 50 nM siRNA against PRKCH or non-targeting control (CTL) siRNA and collected 72 h post treatment. Whole cell lysates were analyzed by Western blot using antibodies against PKC-η, PKC-α, and actin. **(C)** A549 cells transiently expressing PKC-η-DN, PKC-α-DN, and PKC-η-CAT, were infected with *Bt* CDC2721121 at MOI 50 for 1 h, treated with kanamycin (250 μg/ml) for 3 additional hours to suppress extracellular bacterial growth, after which the infected cells were cultured in antibiotic-free media. At 40 h post-infection, the percentage of dead A549 cells was calculated as a ratio of LDH activity in the conditioned media relative to total enzyme activity in the infected cells lysed with 0.1% Triton. The average and standard deviation from three independent experiments are shown. The “^*^” denotes statistical significance (*p* < 0.05) for % cell death in transfected samples compared to infected cells transfected with an empty vector (CTL) for each experimental condition.

We focused our study on PRKCH, which encodes for PKC-η, a novel protein kinase C isoform that requires diacylglycerol (DAG), but not Ca^2+^, for activation. Interestingly, we did not recover any classical Ca^2+^-dependent PKC isoforms (α, β, and γ), which are known to be key regulators of opsonized antigen phagocytosis (Underhill and Ozinsky, 2002). To further examine the differential functions of conventional and novel PKC isoforms in *B. thailandensis* CDC2721121 survival within host cells, we compared the intracellular bacterial load in THP-1 cells siRNA-depleted of PKC-η or PKC-α. Upon downregulation of PKC-η, the average number of intracellular *B. thailandensis* per THP-1 cell dropped by >4-fold compared to control (CTL) cells (Figure 2A). In comparison, downregulation of PKC-α resulted in decreased intracellular bacterial count per host cell by < 2-fold. By Western blot, we observed that siRNA directed toward the PRKCH transcripts was specific, causing the depletion of PKC-η, but not PKC-α protein (Figure 2B). To further test PKC isoform specificity, we examined the induction of host cell death by *B. thailandensis* CDC2721121 infection in lung epithelial A549 cells. This cell model has been previously used to examine *Burkholderia* replication in non-phagocytic cells via formation of polymorphonuclear cells (Wongprompitak et al., 2009; Kunyanee et al., 2016). We transiently expressed dominant-negative mutants of PKC-η (PKC-η-DN) and PKC-α (PKC-α-DN), and a constitutively-active PKC-η (PKC-η-CAT), which lacks the N-terminal regulatory domain, in A549 cells (Soh and Weinstein, 2003). Despite the great data variability originating from transient transfection experiments we observed that cells expressing PKC-η-DN demonstrated reduced host cell death by ~3-fold in response to *Bt* CDC2721121 infection, whereas expression of PKC-α-DN had no significant effect, compared to A549 cells carrying empty vector (CTL) or PKC-η-CAT (Figure 2C). These results indicate that the novel PKC-η isoform plays a more significant role in *B. thailandensis* intracellular survival and induction of host cell death, compared to the conventional PKC-α isoform.

### Intracellular survival of *B. thailandensis* is dependent on MARCKS phosphorylation by PKC-η

The respiratory epithelium and nasal-associated lymphoid tissue serve as primary entry points for inhalational melioidosis (Owen et al., 2009). *Burkholderia* invades host cells via phagocytosis, escapes the membrane-bound phagosome, and disseminates in the host cytoplasm to maintain an intracellular niche (Jones et al., 1996). We investigated a potential role for PKC-η function in the activation of a downstream effector protein, MARCKS, upon *Burkholderia thailandensis* phagocytosis. MARCKS, an ~87 kD protein, functions as an actin cross-linking protein and a signal transducer in PKC-dependent pathways. MARCKS is activated by PKC-catalyzed phosphorylation of a conserved 25 aa basic effector domain (ED). Although MARCKS phosphorylation by the conventional PKC-α has been previously found to facilitate zymosan adherence to macrophage cells and promote phagocytosis (Allen and Aderem, 1995b), the role of novel PKC isoforms on MARCKS function in response to bacterial infection has not yet been investigated.

**Figure 3:**
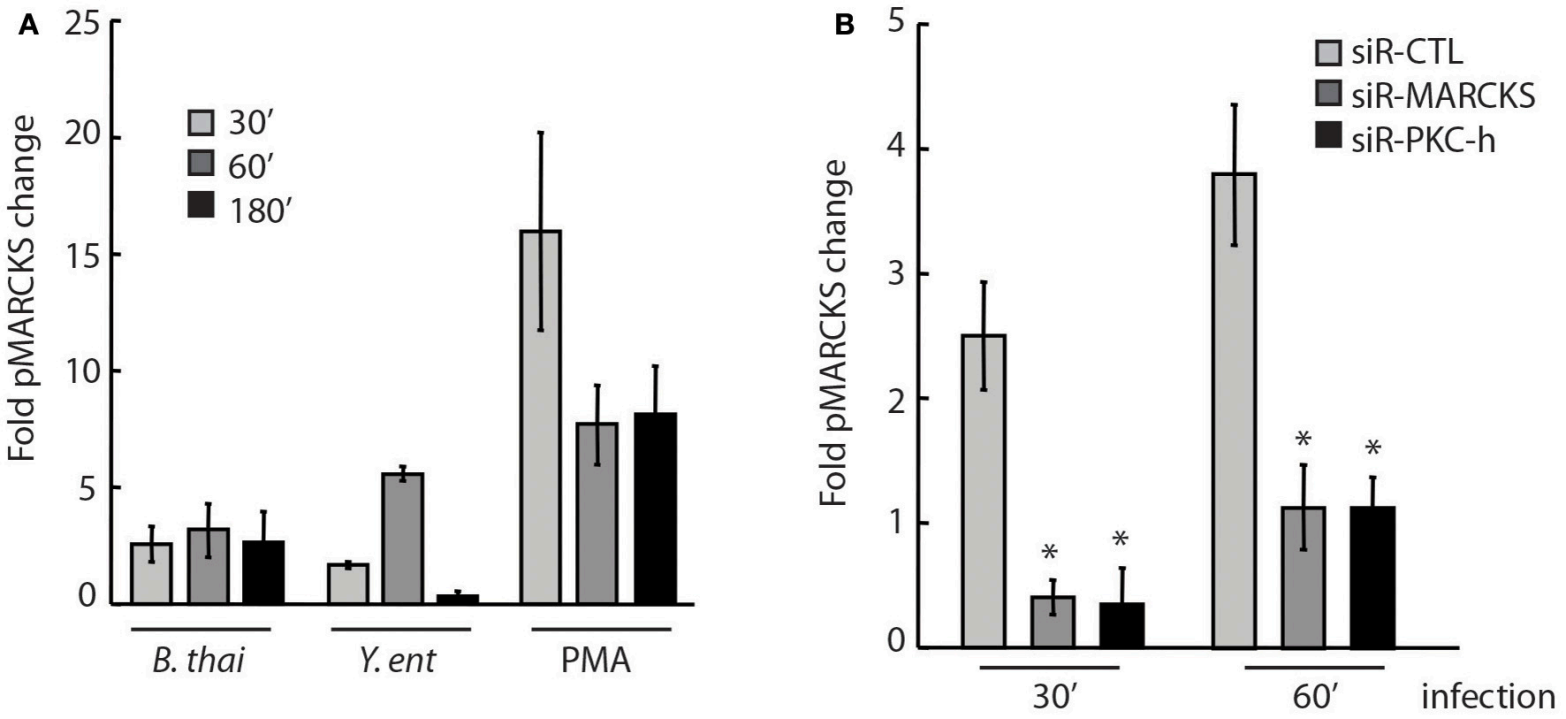
PKC-η is involved in host MARCKS phosphorylation upon bacterial infection (A) A549 cells were infected with *Bt* CDC2721121 at MOI 50 or *Y. enterocolitica* WA at MOI 20. After the indicated times, cells were lysed and levels of phosphorylated Ser152/156 in the MARCKS effector domain (pMARCKS) were quantified using the PathScan ELISA kit and compared to uninfected A549 cells. Cells were also treated with 100 nM PMA as a positive control. In all samples, pMARCKS levels were normalized to the total MARCKS protein levels. The average and standard deviation from three independent experiments are shown. **(B)** A549 cells were treated with 25 nM siRNA against MARCKS, PKC-η, or non-targeting control (CTL) for 72 h prior to infection with *Bt* CDC2721121. At the indicated time, cells were lysed and the levels of pMARCKS were quantified as fold change relative to uninfected A549 cells. The average and standard deviation from three independent experiments are shown. The “^*^” denotes statistical significance (*p* < 0.05) for fold pMARCKS change in samples treated with siRNA to MARCKS or PKC-η, compared to non-targeting siRNA samples (siR-CTL).

We detected a ~2–3-fold increase in phosphorylated MARCKS (pMARCKS) protein levels when normalized to total MARCKS protein in A549 cells infected with *Bt* CDC2721121 for 30, 60, or 180 min, compared to pMARCKS in uninfected cells, using an ELISA specific for MARCKS phosphorylation at Ser175/160 of the ED (Figure 3A). Interestingly, we also observed a time-dependent response to infection with a different Gram-negative pathogen, *Yersinia entercolitica*, in which pMARCKS levels increased from ~1.5-fold at 30 min post-infection to ~5-fold at 60 min, but decreased to basal levels at 180 min. This finding is consistent with the known virulence mechanisms of pathogenic *Yersinia* spp. employing a T3SS to inject Yop effector proteins into the host cells within180 min of infection to inhibit actin polymerization and the host phagocytic response (Zauberman et al., 2006). In comparison, the potent PKC stimulator PMA induced a ~15-fold increase in pMARCKS levels after 30 min and an ~8-fold increase after 60 and 180 min. We also observed that siRNA-mediated knockdown of either MARCKS or PKC-η expression in A549 cells reduced pMARCKS levels by >3-fold in response to 30 or 60 min of infection with *Bt* CDC2721121, compared to host cells treated with non-targeting siRNA (siR-CTL) (Figure 3B). These data indicate that PKC-η participates in MARCKS ED phosphorylation upon infection of lung epithelial cells with Gram-negative bacteria.

**Figure 4:**
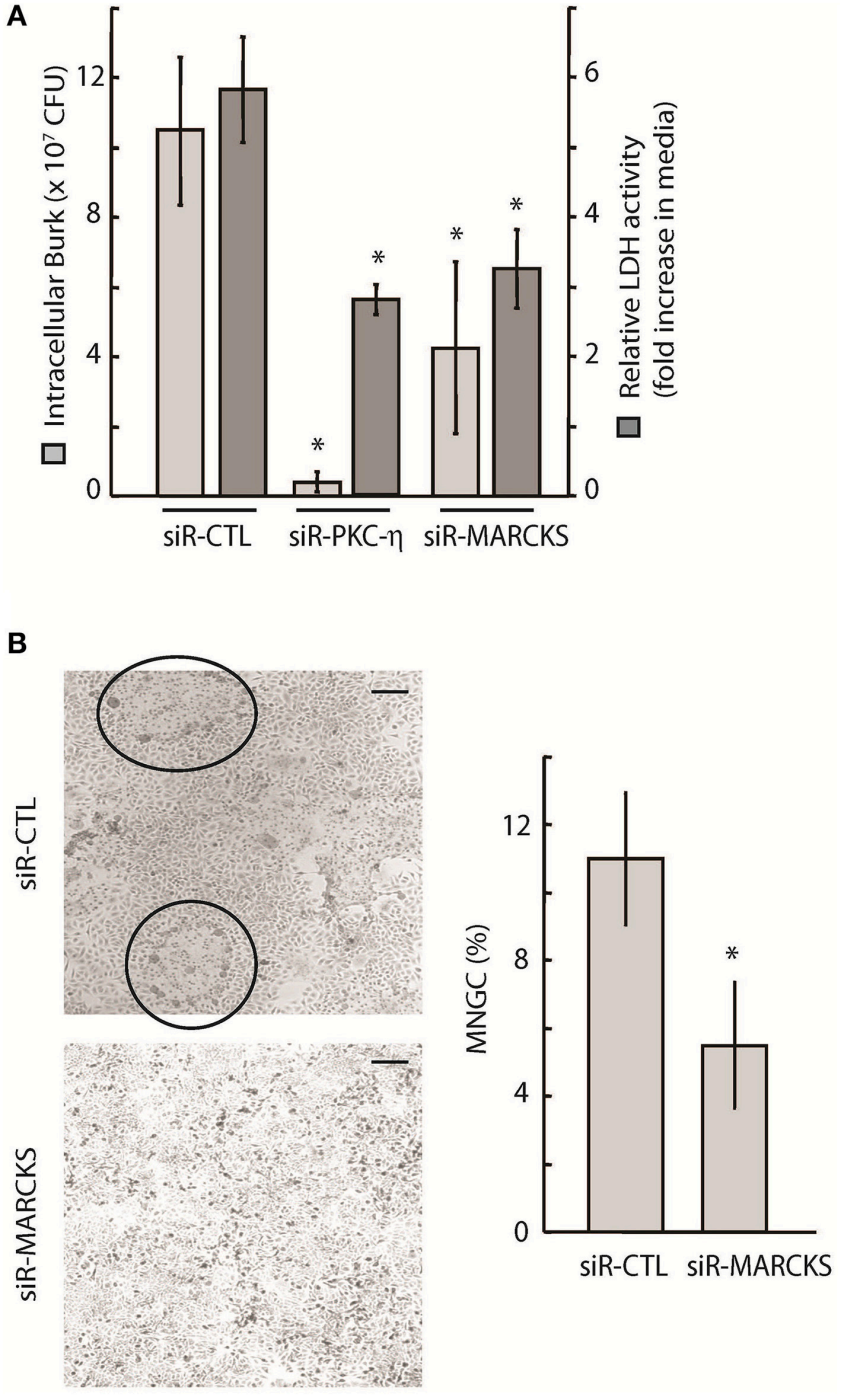
Intracellular survival and spread of pathogenic *Burkholderia* is dependent on PKC-η and MARCKS function. **(A)** A549 cells were treated with 20 nM siRNA targeting MARCKS or PKC-η for 72 h prior to infection with *Bt* CDC2721121 at MOI 50. After 2 h of infection, cells were treated with 250 μg/ml kanamycin for 3 h, and incubated overnight in antibiotic-free media. Cells were collected 24 h post-infection and lysed with 0.1% Triton to release the intracellular bacteria. Colony forming units (CFU) were calculated from limiting dilutions of the cell lysates incubated on nutrient agar for 24 h at 37°C. LDH activity was measured in conditioned media and is presented as fold increase in the 24 vs. 2 h post infection. The average and standard deviation from three independent experiments are shown. The “^*^” denotes statistical significance (*p* < 0.05) for CFU counts and relative LDH activity in samples treated with siRNA to MARCKS or PKC-η, compared to control samples (siR-CTL). **(B)** A549 cells were treated with 20 nM siRNA against MARCKS or with non-targeting (siR-CTL) siRNA 72 h prior to infection with *Bt* CDC2721121 at MOI 50. Cells were fixed 18 h post-infection, stained with Giemsa, and the percentage of MNGC formation was calculated relative to normal cells per field of view. The average and standard deviations were derived from at least 10 fields of view covering an entire 20 mm culture dish. The “^*^” denotes statistical significance (*p* < 0.05) for % MNGC in samples treated with siRNA to MARCKS compared to samples treated with control siRNA (siR-CTL). The scale bar is 60 μM in length.

To further link PKC-η and MARCKS function to bacterial survival within host cells, we demonstrated that PKC-η or MARCKS depletion via siRNA resulted in a ~10-fold and ~2.5-fold reduction, respectively, in the number of viable intracellular *B. thailandensis* in A549 cells following infection for 24 h, compared to cells treated with non-targeting siRNA (siR-CTL) (Figure 4A, light gray bars). This decrease in intracellular *Burkholderia* counts correlated with increased A549 viability of PKC-η– or MARCKS-depleted cells, as measured by a ~2-fold reduction in LDH release (Figure 4A, dark gray bars). We also examined multi-nucleated giant cell (MNGC) formation as a result of *Burkholderia*-induced host cell fusion (Suparak et al., 2005; Galyov et al., 2010). Reduction of gene expression via MARCKS transcript-specific siRNA resulted in ~2-fold less MNGC counts as compared to A549 cells treated with non-targeting siRNA (siR-CTL) (Figure 4B). A statistically marginal difference between controls and experimental samples was observed in most assays where the MARCKS transcript levels were reduced via RNAi. There could be two logical explanations for this phenomenon, (i) native MARCKS transcript levels increase upon bacterial infection (see **Figure 6B**) counteracting the effect of the siRNA on MARCKS transcripts and (ii) MARCKS most probably competes with other PKC-η substrates, including MacMARCKS and proteins referred to as STICKs (substrates that interact with C-kinase) (Chapline et al., 1998). Altogether, our data suggest that MARCKS activation is dependent on PKC-η function, and both proteins are involved in *Burkholderia*-induced host cell pathology following infection.

### PKC-η regulates MARCKS phosphorylation upon engagement of mannose receptors by unopsonized *B. thailandensis*

**Figure 5:**
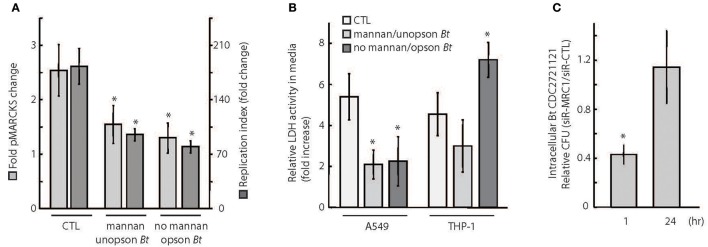
Phagocytosis receptor usage determines the efficiency of intracellular *Burkholderia* proliferation. (A) A549 cells were (1) incubated with 3 mg/ml soluble mannan for 30 min and then infected with unopsonized *Bt* CDC2721121 at MOI 50, (2) not treated with mannan and infected with *Bt* CDC2721121 coated with anti-*B.thailandensis* rabbit serum (opson *Bt*), or (3) not treated with mannan and uninfected (CTL). pMARCKS levels in bacteria-infected cells were quantified and compared to uninfected cells. In parallel, A549 cells were collected after 2 h of infection for determination of intracellular bacterial counts. These cells were treated with 250 μg/ml kanamycin for 3 h to inhibit extracellular bacterial growth and incubated overnight in antibiotic-free media. At 24 h post-infection, a second set of samples were collected, and colony forming units (CFU) were calculated from limiting dilutions of the cell lysates incubated on nutrient agar for 24 h at 37°C. The replication index of intracellular *Bt* CDC2721121 was calculated as the CFU fold change at 24 vs. 2 h post-infection. The average and standard deviation from three independent experiments are shown. The “^*^” denotes statistical significance (*p* < 0.05) for fold change in pMARCKS and replication index in host cells treated with mannan or pathogen coated with serum, compared to CTL samples. **(B)** A549 and THP-1 cells were treated as described in **(A)**, and LDH activity was measured in conditioned media 2 h post infection. After treatment with kanamycin (250 μg/ml) for 3 h, host cells were incubated for 24 h in antibiotic-free media and a second LDH measurement was taken. Relative LDH activity was determined as fold change between the 24 h and 2 h time points post-infection. The average and standard deviation from three independent experiments are shown. The “^*^” denotes statistical significance (*p* < 0.05) for relative LDH activity in experimental samples compared to untreated (CTL) samples. **(C)** A549 cells were treated with 25 nM siRNA targeting MRC1 or with non-targeting (CTL) siRNA and were infected with unopsonized *Bt* CDC2721121 at MOI 50. At 1 h and 24 h post-infection, cells were lysed with 0.1% Triton, and colony forming units (CFU) were calculated from limiting dilutions of the cell lysates incubated on nutrient agar for 24 h at 37°C. The ratio of CFU corresponding to intracellular bacteria from siR-MRC1 treated cells vs. cells treated with non-targeting siRNA is shown for each time point. The average mean and standard deviation from three independent experiments are shown. The “^*^” denotes statistical significance (*p* < 0.05) in number of CFUs between 1 and 24 h.

Pathogen uptake via phagocytosis is initiated by a recognition event between a specific host receptor and microbial cell surface molecules, such as lipopolysaccharide or opsonins (e.g., antibodies) that coat the bacteria. Mannose receptors (MR) play a significant role in the uptake of unopsonized bacteria (Geier and Celli, 2011), but have not yet been found to play a role in microbicidal phagocytic pathways (Astarie-Dequecker et al., 1999). Interestingly, MARCKS was previously reported to function in the phagocytosis of zymosan particles that engage MRs (Carballo et al., 1999). To investigate whether *B. thailandensis CDC2721121* can activate MARCKS phosphorylation via MR engagement, we quantified pMARCKS levels in A549 cells treated with purified mannan to saturate the MRs. A549 cells pre-incubated with mannan exhibited a ~1.5–2-fold decrease in pMARCKS levels following infection with *Bt* CDC2721121, compared to infected cells not treated with mannan (CTL), suggesting that MARCKS phosphorylation is at least partly dependent on MR engagement by the pathogen (Figure 5A, light gray). Alternatively, coating *Bt* CDC2721121 with an opsonin, rabbit anti-*B.th* serum, to block potential interactions between MRs and the pathogen, resulted in ~2-fold reduction of pMARCKS levels in A549 cells. Notably, both conditions decreased the efficiency of *Bt* CDC2721121 infection, reducing the replication index of intracellular *Bt* CDC2721121 in A549 cells by ~2-fold (Figure 5A, dark gray). These data suggest that MARCKS phosphorylation is triggered upon interaction of *Bt* CDC2721121-associated cell surface molecular patterns (eg. mannan polysacharides) with lung epithelial A549 cell receptors, including MR.

Consistent with these observations, both mannan pre-treatment and infection with serum-optimized *Bt* CDC2721121 had a positive effect on A549 host cell viability, as measured by a decrease in extracellular LDH activity (Figure 5B). In contrast, survival of THP-1 cells treated with mannan did not significantly differ from untreated cells (CTL) following infection. This difference in host cell survival between A549 (epithelial) and THP-1 (monocytic) cells upon mannan treatment is likely due to the lower expression levels of MR on monocytes prior to their differentiation into macrophages (Rouleux-Bonnin et al., 1995; Wollenberg et al., 2002). We also observed that serum-opsonized *Bt* CDC2721121 induced a rapid decline in THP-1 viability upon infection, suggesting activation of cell death mechanisms, possibly through Fc receptor (FcR) signaling triggered by antibody opsonins (Underhill and Ozinsky, 2002). Finally, we siRNA-depleted MRC1 (mannose receptor C type 1, CD206) transcripts from A549 cells and observed ~60% reduction in bacterial uptake after 1 h post-infection, relative to host cells treated with non-targeting siRNA (Figure 5C). However, we detected no difference in the intracellular *Bt* CDC2721121 counts between siR-MRC1-treated and control A549 cells after 24 h of infection. These results suggest that mannose receptors facilitate initial *B. thailandensis* uptake but do not significantly contribute to the activation of cellular defense mechanisms that restrict intracellular bacterial growth over time.

### PKC-η facilitates *B. thailandensis* escape into the host cytoplasm

**Figure 6:**
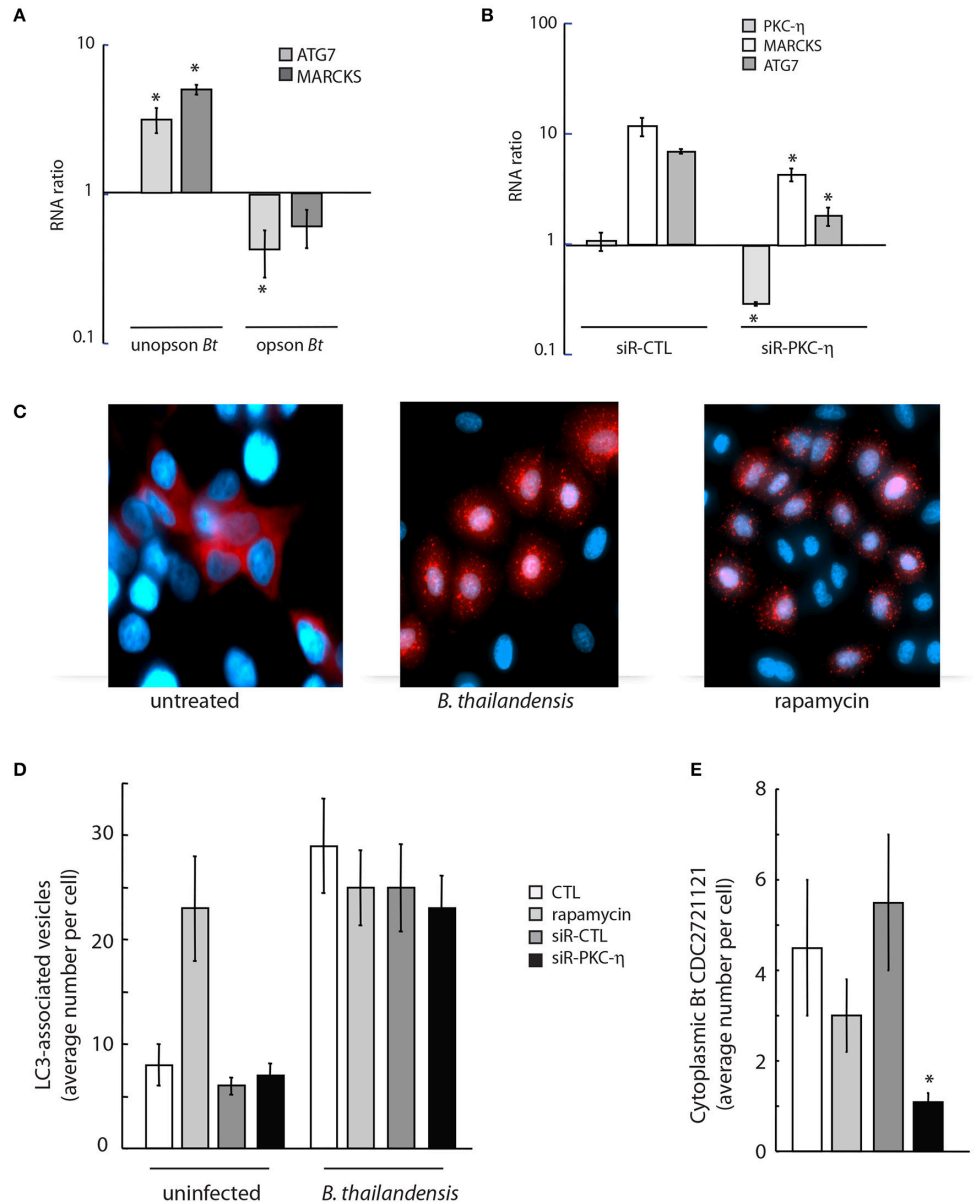
Downregulation of PKC-η gene expression results in reduced cytoplasmic *Burkholderia* load and autophagy initiation. **(A)** A549 cells were infected with unopsonized or serum-coated *Bt* CDC2721121 (opson *Bt*) at MOI 50. Total RNA was isolated 5 h post infection, and transcript levels of ATG7 and MARCKS were quantified in infected vs. uninfected cells. The average and standard deviation from three independent experiments are shown. The “^*^” denotes statistical significance (*p* < 0.05) for RNA expression in infected host cells compared to uninfected samples. **(B)** A549 cells were treated with 20 nM siRNA targeting PKC-η or control siRNA (siR-CTL) for 72 h and then infected with unopsonized *Bt* CDC2721121 at MOI 50. Total RNA was isolated 18 h post infection and changes in gene expression profiles of PKC-η, MARCKS, and ATG7 were quantified relative to uninfected cells. The average and standard deviation from three independent experiments are shown. The “^*^” denotes statistical significance (*p* < 0.05) for RNA expression in samples treated with siR-PKC-η compared to their cognate samples treated with control siRNA. **(C)** A549 cells stably expressing RFP-LC3B were infected with unopsonized *Bt* CDC2721121 at MOI 50 for 6 h or treated with 0.5 μM rapamycin and analyzed by fluorescent microscopy to image LC3-positive autophagosomes. Scale bar, 10 μm. **(D,E)** Quantifying autophagosomes and cytoplasmic *Bt* CDC2721121 in lung epithelial cells with imaging flow cytometry. A549-RFP-LC3B cells were treated with 20 nM siRNA against PKC-η or non-targeting siRNA (siR-CTL) for 72 h *prior* to infection with FITC-labeled *Bt* CDC2721121 at MOI 50. No drug or 10 μM rapamycin was added 3 h before bacterial infection for controls. Cells were treated with 250 μg/ml kanamycin after 2 h of infection and collected 6 h post-infection for analysis. Cells were fixed with 3% paraformaldehyde (pH 6), washed, and then analyzed by Amnis ImageStream^X^. The average number of RFP-LC3 spots was determined by the ratio of total RPF-positive puncta to the number of analyzed single cells. The average number of cytoplasmic bacteria per cell was determined by counting the FITC-positive spots in the green channel and not spots in the red channel divided by the total number of analyzed single cells. The average mean and standard deviation represent statistical analysis of 800 fluorescent positive cells per sample. The “^*^” denotes statistical significance (*p* < 0.05) for the number of cytoplasmic *Burkholderia* in cells depleted of PKC-η compared to untreated control cells (CTL).

To determine whether the opsonization state of *Burkholderia* can affect stimulation of macroautophagy, we quantified expression of ATG7, a regulator of autophagosome formation (Geng and Klionsky, 2008), in response to infection with unopsonized and serum-opsonized *Bt* CDC2721121. We observed ~3-fold increase in ATG7 expression at 5 h post-infection of THP-1 cells with unopsonized *Bt* CDC2721121, but a ~70% decrease in ATG7 levels upon infection with serum-opsonized pathogen, relative to uninfected monocytes (Figure 6A, light gray), suggesting that unopsonized pathogen may preferentially activate autophagosome formation. We also observed a ~5-fold increase in MARCKS transcript levels in THP-1 cells infected with unopsonized *Bt* CDC2721121, whereas there was very little change in MARCKS levels in cells infected with opsonized bacteria (Figure 6A, dark gray). Based on our previous findings that MARCKS function was at least partially regulated by PKC-η (Figure 3B), we also examined a putative role for PKC-η in the regulation of MARCKS and ATG7 gene transcription. We found that both MARCKS and ATG7 transcript levels were reduced ~2- and ~5-fold, respectively, in cells depleted of PKC-η, compared to control siRNA-treated cells (CTL) upon infection with unopsonized *Bt* CDC2721121 (Figure 6B).

To investigate whether PKC-η may be linked to activation of host macroautophagy in response to *B. thailandensis* infection, we monitored autophagosome formation in A549 cells that stably expressed a RFP-tagged fusion to the key autophagy protein LC3/ATG8. We observed formation of RFP-LC3-labeled autophagosome vesicles 6 h after infection with unopsonized *Bt* CDC2721121, similar to treatment with rapamycin, a potent inducer of autophagy flux (Figure 6C). The average number of RFP-LC3-associated autophagosome vesicles in A549 cells increased >3-fold in infected compared to uninfected cells (Figure 6D). We observed that siRNA depletion of PKC-η did not significantly change the number of RFP-LC3-labeled vesicles in infected cells, compared to the untreated (CTL), rapamycin-treated, or siR-CTL-treated controls (Figure 6D). However, we did observe a >75% decrease in intracellular cytoplasmic pathogen that was not associated with RFP-LC3-labeled autophagosome vesicles in PKC-η-depleted A549 cells, compared to untreated (CTL) and siR-CTL treated cells (Figure 6E). Taken together, these results suggest that upon infection with unopsonized *Burkholderia*, PKC-η functions in phagocytic pathways that facilitate bacterial escape into the cytoplasm, which in turn activates host macroautophagy. Here, we demonstrate that inhibition of PKC-η function reduces the events of intracellular bacterial escape into the cytoplasm using the autophagosome flux activation as a reporter marker.

## Discussion

Intracellular pathogens exploit different virulence mechanisms and multiple host factors for efficient invasion, replication, and spread during infection (Hong-Geller and Micheva-Viteva, 2010; Micheva-Viteva et al., 2013; Li et al., 2016). We have performed a RNAi screen and identified 35 genes from the human kinome that play an essential role in the infection of *B. thailandensis*, a close relative of the naturally multidrug-resistant intracellular biothreat pathogen, *B. pseudomallei*. In this study, we have focused on the role of the novel DAG-dependent, Ca^2+^-independent PKC-η isoform in promoting intracellular survival of *Burkholderia* in host cells. The PKC family consists of at least 10 isoforms classified as conventional, novel, and atypical, depending on the requirement for DAG and Ca^2+^ for activation. Atypical DAG- and Ca^2+^-independent PKC isoforms (e.g., PKC- ζ) have also previously been implicated in phagosomal escape and intracellular survival of *Listeria* and *Plasmodium* sporozoites, suggesting that Ca^2+^-independent novel and atypical PKC isoforms can mediate intracellular pathogen infection (Prudencio et al., 2008; Jiwani et al., 2012). Conventional DAG- and Ca^2+^-dependent PKC isoforms (e.g., PKC-α) regulate actin cytoskeleton dynamics and antimicrobial immune responses, but were not identified in our screen. We had further observed that a dominant-negative variant of PKC-α did not significantly affect host cell survival upon *B. thailandensis* infection. Specific roles for PKC isoforms in CD40 signaling and macrophage infection by *Leishmania major* have been reported (Sudan et al., 2012). Infection of macrophages with *L. major* was found to enhance PKCδ/ζ/λ–dependent activation of ERK1/2 phosphorylation and *Leishmania* growth, but impair the functions of PKCα/β/ε responsible for p38MAPK phosphorylation and parasite killing, thus defining differential roles for PKC isoforms in immune homeostasis.

This study links pathogen opsonization with the divergent functions of PKC isoforms. Our results show that infection with unopsonized *B. thailandensis* specifically activates PKC-η regulation of MARCKS function on both transcriptional (expression) and post-transcriptional (phosphorylation) levels to enable pathogen survival within the host. We observed that inhibition of PKC-η or MARCKS function by siRNA or functional mutant expression reduced the efficiency of *Bt* CDC2721121 infection in A549 cells. Infection with *Y. enterocolitica* also led to an increase in MARCKS phosphorylation, suggesting that multiple Gram-negative pathogens can activate the PKC-η/MARCKS pathway during infection. MARCKS, a phosphorylation target of PKC, has itself been previously linked to membrane trafficking and actin dynamics. Receptor-dependent PKC stimulation regulates trafficking of MARCKS between the plasma membrane, LAMP-1–positive lysosomes (Allen and Aderem, 1995a), and mucin granules (Li et al., 2001). Formation of F-actin-rich lamellipodia is dependent on the dephosphorylation and translocation of MARCKS to lipid rafts, causing the recruitment of β-actin to membrane microdomains (Yamaguchi et al., 2009). Moreover, a 24 aa peptide derived from the N-terminus of MARCKS was shown to block mucus secretion in a mouse model of asthma, further implicating MARCKS function in actin-mediated secretion (Singer et al., 2004).

Host cell surface receptors, such as MR or FcR, can mediate bacterial uptake via recognition of mannan or antibody opsonins, respectively, on the pathogen cell surface. Interestingly, macrophages and fibroblasts isolated from MARCKS-knockout mouse embryos exhibited a decrease in only the MR-mediated uptake of zymosan-coated particles compared to wild type cells, thus linking MR and MARCKS functions in phagocytosis (Underhill et al., 1998; Carballo et al., 1999). We find that either saturation of host mannose receptors with mannan or coating of pathogen with anti-sera to block surface interactions and re-direct phagocytic receptor usage leads to a decrease in the replication index of *B. thailandensis* in A549 cells, suggesting that MR at least partly mediates the uptake and intracellular replication of unopsonized *B. thailandensis*. We also demonstrate that PKC-η is required for the intracellular survival of unopsonized *B. thailandensis* through the MR-MARCKS pathway.

**Figure 7:**
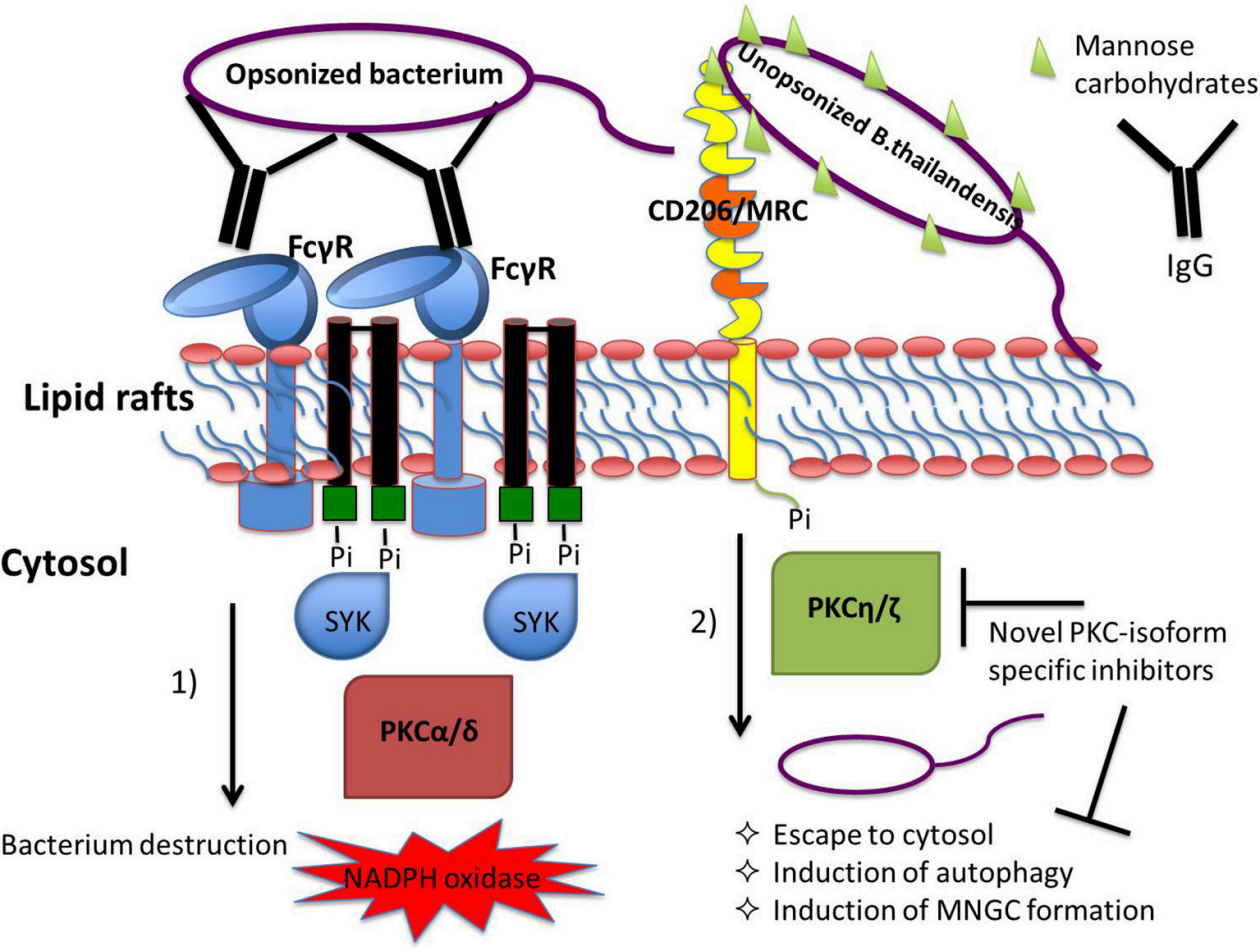
Coating of bacteria with opsonin determines receptor usage and the involvement of protein kinase C (PKC) isoforms in the regulation of phagocytosis-associated cell responses. Immunoglobulin G-opsonized bacteria triggers FcγRs-mediated phagocytosis engaging classical PKC isoform signaling pathways leading to pro-inflammatory cell responses, including the respiratory burst leading to efficient killing of bacteria within the lysosomes (Breton and Descoteaux, 2000; Dekker et al., 2000; Sudan et al., 2012). Interaction of bacterial glycoproteins with the host cell mannose receptor C-type 1, MRC1, stimulates signaling pathways regulated by the Ca^2+^-independent PKC isoforms resulting in escape of bacteria from the phagosome into the cytosol. Our hypothesis is that PKC inhibitors with selectivity to the novel, Ca^2+^-independent isoforms have the potential to act as host-directed antimicrobials blocking pathogen escape from phagosome destruction.

The results presented here further enhance our understanding of the molecular machinery that regulates phagocytosis (Figure 7). It has been previously demonstrated that the PKC isoforms required for phagocytosis appear to be different in monocytes and mature macrophages. In monocytes FcγRI stimulation was found to increase PKC activity corresponding to the membrane translocation of the Ca ^2+^-independent PKC isoforms δ, ε, and ζ while FcγRI stimulation in monocyte-differentiated macrophages involved PKC activity that corresponded to the membrane translocation of the PKC isoforms α, β, and γ (Melendez et al., 1999). To date no particular role has been assigned to the novel isoform PKC-η in FcγRI signaling. We observed that infection of undifferentiated monocytic THP-1 cells with bacteria coated with anti-*B. thailandensis* serum resulted in reduced expression of ATG7 and MARCKS, genes we found to be at least partially regulated by the Ca^2+^-independent PKC-η (Figure 6). Our data suggest that this novel isoform has a stronger effect on phagocytic pathways initiated by receptors recognizing pathogen associated molecular patterns in contrast to phagocytosis stimulated by FcγR intearction with IgG opsonized bacteria.

In response to pathogen invasion, the host will attempt to restrict intracellular bacterial proliferation by mounting defense mechanisms such as macroautophagy (Romao et al., 2013; Munz, 2014), a process by which cytoplasmic constituents and dysfunctional organelles are recycled in eukaryotic cells (Choy and Roy, 2013; Pareja and Colombo, 2013). While autophagy can effectively inhibit intracellular survival of some bacteria (Gutierrez et al., 2004; Nakagawa et al., 2004), *Burkholderia* species have been shown to subvert this host defense mechanism to establish persistent infection (Colombo et al., 2006; Ogawa and Sasakawa, 2006; Rodrigues et al., 2007). Induction of autophagy in murine macrophages can suppress the intracellular survival of pathogenic *B. pseudomallei*, which in turn, induces the expression of the Type 3 secretion system (T3SS) effector BopA to evade autophagy (Cullinane et al., 2008). We observed that expression of ATG7, a mediator of autophagy, is upregulated upon infection with unopsonized *B. thailandensis*, suggesting that uptake of unopsonized pathogen can lead to phagosomal escape that triggers autophagy. Furthermore, siRNA-mediated depletion of PKC-η significantly inhibited ATG7 gene expression levels. Finally, our finding that the counts of *B. thailandensis* not associated with phagosomes was reduced upon PKC-η depletion, whereas phagosome vesicle formation remained unchanged, suggests that PKC-η plays an indirect role in the activation of autophagosome flux, likely by facilitating phagocytic pathways that promote *Burkholderia* escape into the cytoplasm.

All together, our results support a model in which PKC-η/MARCKS signaling can promote intracellular survival of unopsonized *B. thailandensis*, at least partly through activation of MR. Isoform-specific, PKC-targeted immunotherapy based on *in vivo* gene silencing has been reported to protect susceptible BALB/c mice from *L. major* infection (Sudan et al., 2012). Highly specific small molecule inhibitors against the novel PKC-η/ζ/ε isoforms, with ~100-fold selectivity over the conventional PKC α/β isoforms, have been identified (van Eis et al., 2011) and could be tested as potential modulators of cellular innate defense against intracellular pathogens. Thus, small molecule inhibitors with selectivity to the novel PKC isoform- group may have the potential to act as host-directed antimicrobials blocking pathogen escape from host-mediated destruction. Such agents may also contribute to the reduction of antimicrobial resistance to conventional antibiotics (Czyz et al., 2014).

## Author contributions

Conceived and designed the experiments: SM and EH. Performed the experiments: SM, YS, KG, and TW. Analyzed the data: SM and EH. Wrote the paper: SM and EH.

### Conflict of interest statement

The authors declare that the research was conducted in the absence of any commercial or financial relationships that could be construed as a potential conflict of interest.
