# Supplementary material for: PKC-η-MARCKS Signaling Promotes Intracellular Survival of Unopsonized Burkholderia thailandensis
Source: Front Cell Infect Microbiol. 2017 Jun 7;7:231. doi: 10.3389/fcimb.2017.00231 (PMC5461351; doi:10.3389/fcimb.2017.00231)
Supplement: Supplementary file 1 [file DataSheet1.DOCX]

PKC-η-MARCKS Signaling Promotes Intracellular Survival of Unopsonized *Burkholderia*

Sofiya N. Micheva-Viteva^1^, Yulin Shou^1^, Kumkum Ganguly^1^, Terry H. Wu^2^,

and Elizabeth Hong-Geller^1,#^

^1^Bioscience Division, Los Alamos National Laboratory, Los Alamos, NM 87544

^2^Center for Infectious Disease & Immunity and Department of Internal Medicine, University of New Mexico Health Sciences Center, Albuquerque, NM 87131

**Supplementary Materials and Methods**

**High-throughput siRNA screen of *B. thailandensis*-infected human monocytes** –THP-1 cells (50000 per well) were transfected with the Human siGENOME siRNA SMARTpool Library for Protein Kinases (718 genes, Thermo Scientific/Dharmacon, Pittsburgh, PA) consisting of four independent inhibitory sequences per target gene at a final concentration of 50 nM per reaction in a 96-well format using Dharmafect4 transfection reagent (Thermo Scientific). Briefly, 1µl of 5µM siRNA was diluted in 10µl serum-free RPMI and combined with 10µl serum-free RPMI containing 0.3µl Dharmafect4. The transfection complex was incubated at room temperature for 20 min and added to 80µl of THP-1 cells in complete media (RPMI/10% FBS). THP-1 cells were infected with *B. thailandensis* DW503-GFP at MOI 50, 72h post-transfection with siRNA. Infection was terminated 2h post-bacterial exposure by adding fresh RPMI/10%FBS media containing gentamycin to a final concentration of 250µg/ml. Trypan blue (80µg/ml) was added to the infected THP-1 cells to quench GFP fluorescence from the extracellular bacteria. 100µl of each sample were transferred to a new 96-well plate 4h post-infection, and the percentage of GFP-positive THP-1 cells was determined using the LSRII flow cytometer platform in HTS mode (BD Biosciences, San Jose, CA). Fresh antibiotic-free media was added to the remaining THP-1 cells and analyzed 20h later. 10,000 cells were acquired at 488 nm and 585/42 nm emission channels. The population of GFP-positive THP-1 cells was selected relative to the population of uninfected THP-1 cells treated with non-targeting control siRNA and processed in parallel.

**HTS data analysis and hit identification** *-* The % GFP-positive THP-1 cells at 24h post infection with DW503-GFP was determined using the BD FACSDiva version 6.0 software and plotted against the corresponding siRNA. The assay cut-off was established as two standard deviations below the average percentage of GFP-positive THP-1 cells measured in negative control samples, including infected cells treated solely with transfection reagent or with non-targeting siRNA. Positive controls included THP-1 cells treated with cytochalasin D (1µM) and the ABL small molecule inhibitor PD180970 (3µM), which exhibited reduced uptake of DW503-GFP. A standard z-score was calculated based on the average values and standard deviations from the negative and positive control samples collected from all experimental plates: z = (x-µ)/σ, where x is % GFP-positive THP-1 cells from a single well, µ is the mean reporter signal calculated for the negative control samples and σ is the standard deviation of all control samples versus the medium of the assay reads. Genes that exhibited a reduction in the GFP-positive THP-1 population with a z <-2 upon siRNA silencing in two independent experiments were considered to be hits.

**Supplementary Table 1**. Flow cytometry-based RNAi HTS discovered host kinases that support *B.thailandensis* DW503-GFP intracellular replication within THP-1 cells. The percentage of GFP-positive THP-1 cells was determined using the LSRII flow cytometer platform in HTS mode at 4 and 24h post-infection. The replication index of intracellular bacteria was determined as the ratio of GFP-positive THP-1 cells at 24h relative to 4h post-infection for each HTS run. NTC, non-targeting siRNA; positive controls are shown in green, the initial gene hits are highlighted in red, and genes encoding for classic PKC are highlighted in yellow. This is an example of the data from the screen for a representative plate (Plate 8).

| HTS 1 | | HTS 2 | | Plate 8 | | | | |  | |
| --- | --- | --- | --- | --- | --- | --- | --- | --- | --- | --- |
| GFP-positive THP1 cells% | | | |  |  | Replication Index, RI | | | Z' factor for 24h pi | |
| 4h | 24h | 4h | 24h | gene | Well | HTS1 | | HTS2 | HTS1 | HTS2 |
| 3.8 | 8.2 | 3 | 6.5 | NTC | A1 | 2.16 | | 2.17 | 0.024691 | 0 |
| 5.1 | 10.1 | 2.9 | 9.9 | MELK | A2 | 1.98 | | 3.41 | 0.806584 | 1.954023 |
| 1.5 | 2.3 | 3.2 | 3.2 | PRKCH | A3 | 1.53 | | 1.00 | -2.40329 | -1.89655 |
| 0.4 | 1.2 | 1.3 | 1.8 | PAK3 | A4 | 3.00 | | 1.38 | -2.85597 | -2.70115 |
| 2.7 | 6.6 | 3.7 | 7.5 | PFKFB1 | A5 | 2.44 | | 2.03 | -0.63374 | 0.574713 |
| 1.1 | 4.1 | 2.6 | 4.7 | INSRR | A6 | 3.73 | | 1.81 | -1.66255 | -1.03448 |
| 4.3 | 7.2 | 2.7 | 9.1 | DLG2 | A7 | 1.67 | | 3.37 | -0.38683 | 1.494253 |
| 2.5 | 4.1 | 2.9 | 4.3 | PIP5K3 | A8 | 1.64 | | 1.48 | -1.66255 | -1.26437 |
| 3.1 | 8.2 | 2.7 | 9.3 | BMPR1A | A9 | 2.65 | | 3.44 | 0.024691 | 1.609195 |
| 2.1 | 7.3 | 3.8 | 8.8 | PRPS1L1 | A10 | 3.48 | | 2.32 | -0.34568 | 1.321839 |
| 3.6 | 6.1 | 2.7 | 5.8 | IGF2R | A11 | 1.69 | | 2.15 | -0.83951 | -0.4023 |
| 0.5 | 0.6 | 1.5 | 0.2 | PD180970 (3µM) | A12 | 1.20 | | 0.13 | -2.69136 | -3.62069 |
| 3.6 | 8.9 | 2.9 | 6.6 | NTC | B1 | 2.47 | | 2.28 | 0.312757 | 0.057471 |
| 2.6 | 6.2 | 2.6 | 8.3 | TGFBR3 | B2 | 2.38 | | 3.19 | -0.79835 | 1.034483 |
| 2.5 | 7.6 | 2.5 | 7.1 | UMP-CMPK | B3 | 3.04 | | 2.84 | -0.22222 | 0.344828 |
| 2.4 | 6.1 | 6.3 | 6.1 | CHKA | B4 | 2.54 | | 0.97 | -0.83951 | -0.22989 |
| 2.6 | 5.5 | 3.1 | 5.1 | WNK4 | B5 | 2.12 | | 1.65 | -1.08642 | -0.8046 |
| 3.5 | 5.7 | 4.7 | 5.3 | RPS6KA1 | B6 | 1.63 | | 1.13 | -1.00412 | -0.68966 |
| 2.8 | 9.5 | 3.8 | 7.5 | IHPK3 | B7 | 3.39 | | 1.97 | 0.559671 | 0.574713 |
| 2.3 | 7.3 | 3.3 | 6.3 | BMX | B8 | 3.17 | | 1.91 | -0.34568 | -0.11494 |
| 2.5 | 6.5 | 2.1 | 4.5 | CLK4 | B9 | 2.60 | | 2.14 | -0.6749 | -1.14943 |
| 1.8 | 4.5 | 3.2 | 4.2 | PAK2 | B10 | 2.50 | | 1.31 | -1.49794 | -1.32184 |
| 3.8 | 6.3 | 2.3 | 6.8 | LOC390226 | B11 | 1.66 | | 2.96 | -0.7572 | 0.172414 |
| 1.1 | 2.5 | 1 | 0.7 | PD180970 (3µM) | B12 | 2.27 | | 0.70 | -2.32099 | -3.33333 |
| 3.9 | 8.4 | 3.8 | 7.6 | NTC | C1 | 2.15 | | 2.00 | 0.106996 | 0.632184 |
| 2.4 | 5.6 | 1.4 | 8.8 | CSNK1A1 | C2 | 2.33 | | 6.29 | -1.04527 | 1.321839 |
| 2.3 | 8.2 | 3.3 | 6.9 | NME2 | C3 | 3.57 | | 2.09 | 0.024691 | 0.229885 |
| 1.6 | 4.1 | 4.2 | 7.1 | MAK | C4 | 2.56 | | 1.69 | -1.66255 | 0.344828 |
| 3.2 | 7.1 | 3.9 | 4.7 | PIK3CD | C5 | 2.22 | | 1.21 | -0.42798 | -1.03448 |
| 1.6 | 6.3 | 5.8 | 5.1 | MAP3K6 | C6 | 3.94 | | 0.88 | -0.7572 | -0.8046 |
| 1.8 | 5.4 | 4.3 | 7.2 | BRDT | C7 | 3.00 | | 1.67 | -1.12757 | 0.402299 |
| 1.8 | 9.2 | 2.7 | 8.5 | EIF2AK4 | C8 | 5.11 | | 3.15 | 0.436214 | 1.149425 |
| 1.6 | 4.1 | 3 | 7.3 | NME6 | C9 | 2.56 | | 2.43 | -1.66255 | 0.45977 |
| 1.1 | 3.7 | 2.1 | 5.9 | DYRK1A | C10 | 3.36 | | 2.81 | -1.82716 | -0.34483 |
| 0.6 | 2.1 | 2.6 | 4.6 | CAMK4 | C11 | 3.50 | | 1.77 | -2.4856 | -1.09195 |
| 0.1 | 0.5 | 0.5 | 0.5 | CytoD | C12 | 5.00 | | 1.00 | -3.14403 | -3.44828 |
| 4.4 | 6.4 | 4.5 | 6.2 | NTC | D1 | 1.45 | | 1.38 | -0.71605 | -0.17241 |
| 3.3 | 6.2 | 4.2 | 5.2 | JIK | D2 | 1.88 | | 1.24 | -0.79835 | -0.74713 |
| 2.1 | 4.1 | 2.8 | 5 | PKLR | D3 | 1.95 | | 1.79 | -1.66255 | -0.86207 |
| 0.9 | 1 | 3.8 | 3.9 | PAK7 | D4 | 1.22 | | 1.03 | -2.52675 | -1.49425 |
| 2 | 6.5 | 3.4 | 8.3 | VRK1 | D5 | 3.25 | | 2.44 | -0.6749 | 1.034483 |
| 1.6 | 5.1 | 3.3 | 6.2 | EPHA6 | D6 | 3.19 | | 1.88 | -1.25103 | -0.17241 |
| 2.6 | 8.2 | 2.4 | 5.8 | GUCY2C | D7 | 3.15 | | 2.42 | 0.024691 | -0.4023 |
| 1.9 | 7.4 | 2.8 | 6.9 | PNCK | D8 | 3.89 | | 2.46 | -0.30453 | 0.229885 |
| 1.3 | 8.1 | 3.2 | 6.5 | VRK3 | D9 | 6.23 | | 2.03 | -0.01646 | 0 |
| 2.6 | 6.3 | 1.4 | 5.4 | PDGFRA | D10 | 2.42 | | 3.86 | -0.7572 | -0.63218 |
| 2.7 | 6.6 | 2.2 | 7.7 | DDR1 | D11 | 2.44 | | 3.50 | -0.63374 | 0.689655 |
| 0.4 | 0.6 | 0.7 | 0.6 | CytoD | D12 | 1.50 | | 0.86 | -3.10288 | -3.3908 |
| 4.1 | 12.6 | 1.9 | 6.9 | NTC | E1 | 3.07 | | 3.63 | 1.835391 | 0.229885 |
| 2.3 | 4.6 | 3.3 | 4.8 | PTK9L | E2 | 2.00 | | 1.45 | -1.45679 | -0.97701 |
| 0.4 | 3.2 | 1.5 | 3.6 | PIK3C2A | E3 | 8.00 | | 2.40 | -2.03292 | -1.66667 |
| 1 | 1.9 | 2.7 | 4.5 | STK32A | E4 | 1.90 | | 1.67 | -2.5679 | -1.14943 |
| 2.4 | 6.3 | 3.2 | 6.4 | GTF2H1 | E5 | 2.63 | | 2.00 | -0.7572 | -0.05747 |
| 3.9 | 7.3 | 2.2 | 7.1 | TOPK | E6 | 1.87 | | 3.23 | -0.34568 | 0.344828 |
| 2.9 | 9.1 | 2.6 | 6.5 | ZAK | E7 | 3.14 | | 2.50 | 0.395062 | 0 |
| 2.9 | 6.3 | 5.5 | 6.8 | PDK4 | E8 | 2.17 | | 1.24 | -0.7572 | 0.172414 |
| 3.7 | 9.8 | 4.8 | 7.1 | CHUK | E9 | 2.65 | | 1.48 | 0.683128 | 0.344828 |
| 4.2 | 7.3 | 1.7 | 5.5 | TPK1 | E10 | 1.74 | | 3.24 | -0.34568 | -0.57471 |
| 3.2 | 6.4 | 2.4 | 7.3 | C14ORF20 | E11 | 2.00 | | 3.04 | -0.71605 | 0.45977 |
| 1.1 | 2.7 | 1.2 | 3.3 | siR_Abl | E12 | 2.45 | | 2.75 | -2.23868 | -1.83908 |
| 3.7 | 7.1 | 2.1 | 6.6 | NTC | F1 | 1.92 | | 3.14 | -0.42798 | 0.057471 |
| 3.5 | 6.1 | 3.6 | 8.7 | PIM3 | F2 | 1.74 | | 2.42 | -0.83951 | 1.264368 |
| 2.3 | 4.1 | 6.7 | 6.3 | LMTK3 | F3 | 1.78 | | 0.94 | -1.66255 | -0.11494 |
| 2.8 | 8.5 | 5.4 | 6.6 | CHEK2 | F4 | 3.04 | | 1.22 | 0.148148 | 0.057471 |
| 3.2 | 5.8 | 8.5 | 7.2 | JAK1 | F5 | 1.81 | | 0.85 | -0.96296 | 0.402299 |
| 2 | 7.2 | 2.7 | 5.3 | EIF2AK3 | F6 | 3.60 | | 1.96 | -0.38683 | -0.68966 |
| 0.7 | 3.4 | 2.2 | 7.4 | PIK3C3 | F7 | 4.86 | | 3.36 | -1.95062 | 0.517241 |
| 3.8 | 6.4 | 2.7 | 5.2 | ROCK2 | F8 | 1.68 | | 1.93 | -0.71605 | -0.74713 |
| 1.1 | 2.8 | 2.5 | 4.6 | PIK3C2B | F9 | 2.55 | | 1.84 | -2.19753 | -1.09195 |
| 3.7 | 7.4 | 2.2 | 4.9 | SGK | F10 | 2.00 | | 2.23 | -0.30453 | -0.91954 |
| 3.4 | 5.8 | 5.5 | 4.9 | CLK3 | F11 | 1.71 | | 0.89 | -0.96296 | -0.91954 |
| 0.2 | 0.3 | 0.7 | 1.9 | siR_Abl | F12 | 1.50 | | 2.71 | -3.22634 | -2.64368 |
| 3.7 | 5.4 | 4.3 | 6.4 | NTC | G1 | 1.46 | | 1.49 | -1.12757 | -0.05747 |
| 3.3 | 6.5 | 2.7 | 7.5 | PXK | G2 | 1.97 | | 2.78 | -0.6749 | 0.574713 |
| 3.6 | 5.7 | 6 | 9.7 | HUNK | G3 | 1.58 | | 1.62 | -1.00412 | 1.83908 |
| 0.9 | 3.8 | 3.4 | 5.7 | MAP4K1 | G4 | 4.22 | | 1.68 | -1.78601 | -0.45977 |
| 4.5 | 11.5 | 3.5 | 5.7 | PCTK2 | G5 | 2.56 | | 1.63 | 1.382716 | -0.45977 |
| 2.3 | 4.8 | 2.4 | 4.4 | INSR | G6 | 2.09 | | 1.83 | -1.37449 | -1.2069 |
| 3 | 5.1 | 5.2 | 7.1 | HIPK4 | G7 | 1.70 | | 1.37 | -1.25103 | 0.344828 |
| 4.4 | 6.7 | 2.2 | 8.4 | SIK2 | G8 | 1.52 | | 3.82 | -0.59259 | 1.091954 |
| 2.6 | 5.5 | 2 | 7.3 | MET | G9 | 2.12 | | 3.65 | -1.08642 | 0.45977 |
| 5.2 | 6.8 | 6.2 | 8.9 | FYN | G10 | 1.31 | | 1.44 | -0.55144 | 1.37931 |
| 2.1 | 5 | 2.8 | 7.9 | DKFZP434C131 | G11 | 2.38 | | 2.82 | -1.29218 | 0.804598 |
| 1.2 | 6.6 | 5.1 | 4.4 | NTC | H1 | 5.50 | | 0.86 | -0.63374 | -1.2069 |
| 0.8 | 0.1 | 0.4 | 0.6 | siR_AKT1 | G12 | 0.13 | | 1.50 | -3.30864 | -3.3908 |
| 2.4 | 8.7 | 3.6 | 6.6 | SAST | H2 | 3.63 | | 1.83 | 0.230453 | 0.057471 |
| 2.1 | 4.3 | 1.8 | 5.5 | NEK2 | H3 | 2.05 | | 3.06 | -1.58025 | -0.57471 |
| 1.9 | 8.9 | 4.9 | 4.5 | MAPKAPK5 | H4 | 4.68 | | 0.92 | 0.312757 | -1.14943 |
| 2.9 | 6.2 | 5 | 4.2 | NRK | H5 | 2.14 | | 0.84 | -0.79835 | -1.32184 |
| 2.8 | 7.5 | 8.9 | 6.3 | PFKFB2 | H6 | 2.68 | | 0.71 | -0.26337 | -0.11494 |
| 2.1 | 6.7 | 2.2 | 5.2 | MARK4 | H7 | 3.19 | | 2.36 | -0.59259 | -0.74713 |
| 2.2 | 4.2 | 5.5 | 2.6 | EPHB1 | H8 | 1.91 | | 0.47 | -1.6214 | -2.24138 |
| 2.9 | 8.5 | 3.6 | 5.8 | MAGI-3 | H9 | 2.93 | | 1.61 | 0.148148 | -0.4023 |
| 2.3 | 6.6 | 2.4 | 4.8 | SSTK | H10 | 2.87 | | 2.00 | -0.63374 | -0.97701 |
| 3.4 | 6.2 | 3.2 | 5.8 | IKBKG | H11 | 1.82 | | 1.81 | -0.79835 | -0.4023 |
| 1.5 | 2.7 | 2.5 | 3.3 | siR_AKT1 | H12 | 1.80 | | 1.32 | -2.23868 | -1.83908 |
| HTS 1 | | HTS 2 | | Plate 2 | | | | | | |
| GFP-positive THP1 cells% | | | |  |  | | Replic Index, RI | | Z' factor for 24h | |
| 4h | 24h | 4h | 24h | gene | Well | | HTS1 | HTS2 | HTS1 | HTS2 |
| 2.8 | 6.1 | 4.9 | 8.9 | NTC | A1 | | 2.18 | 2.10 | 0.984962 | -1.36667 |
| 3 | 4.3 | 5.4 | 8.3 | PIK3CA | A2 | | 1.43 | 1.54 | -0.36842 | -0.56667 |
| 2.3 | 3.6 | 4.6 | 10.2 | KIAA1804 | A3 | | 1.57 | 2.22 | -0.89474 | 0.066667 |
| 2.5 | 4.3 | 4.4 | 10.3 | NAGK | A4 | | 1.72 | 2.34 | -0.36842 | 0.1 |
| 2.1 | 4.8 | 4 | 7.2 | IRAK1 | A5 | | 2.29 | 1.80 | 0.007519 | -0.93333 |
| 2.5 | 4.9 | 4.3 | 10.5 | EEF2K | A6 | | 1.96 | 2.44 | 0.082707 | 0.166667 |
| 2.8 | 7.1 | 5.2 | 8.5 | ANKK1 | A7 | | 2.54 | 1.63 | 1.736842 | -0.5 |
| 3.1 | 4.6 | 5.6 | 7.7 | MAP3K5 | A8 | | 1.48 | 1.38 | -0.14286 | -0.76667 |
| 2.8 | 4.8 | 5.1 | 8.2 | PRKAA1 | A9 | | 1.71 | 1.61 | 0.007519 | -0.6 |
| 1.6 | 3.4 | 2.9 | 6.2 | RPS6KA4 | A10 | | 2.13 | 2.14 | -1.04511 | -1.26667 |
| 2.5 | 1.3 | 4.8 | 2.9 | CALM1 | A11 | | 0.52 | 0.60 | -2.62406 | -2.36667 |
| 1 | 1.3 | 2.1 | 1.7 | PD180970 (3µM) | A12 | | 1.30 | 0.81 | -2.62406 | -2.76667 |
| 2.3 | 4 | 4.3 | 6.1 | NTC | B1 | | 1.74 | 1.42 | -0.59398 | -1.3 |
| 3 | 4.6 | 6 | 7.8 | FLJ32685 | B2 | | 1.53 | 1.30 | -0.14286 | -0.73333 |
| 4 | 4.1 | 7.2 | 9.4 | ITPKC | B3 | | 1.03 | 1.31 | -0.5188 | -0.2 |
| 0.3 | 0.2 | 0.9 | 1.1 | EPHA5 | B4 | | 0.67 | 1.22 | -3.45113 | -2.96667 |
| 2.9 | 4.1 | 5.4 | 7.3 | MLCK | B5 | | 1.41 | 1.35 | -0.5188 | -0.9 |
| 2.2 | 3.4 | 4.2 | 9.4 | PSKH1 | B6 | | 1.55 | 2.24 | -1.04511 | -0.2 |
| 2.9 | 0.3 | 6.1 | 1.2 | AURKC | B7 | | 0.10 | 0.20 | -3.37594 | -2.93333 |
| 2.9 | 1.5 | 5.3 | 2.7 | PRKCE | B8 | | 0.52 | 0.51 | -2.47368 | -2.43333 |
| 1.8 | 1.9 | 3.8 | 5.1 | AK1 | B9 | | 1.06 | 1.34 | -2.17293 | -1.63333 |
| 2.5 | 3 | 4.9 | 7.5 | TRPM6 | B10 | | 1.20 | 1.53 | -1.34586 | -0.83333 |
| 2.4 | 3.3 | 4.6 | 8.5 | CLK1 | B11 | | 1.38 | 1.85 | -1.1203 | -0.5 |
| 1.2 | 1.4 | 2.1 | 1.9 | PD180970 (3µM) | B12 | | 1.17 | 0.90 | -2.54887 | -2.7 |
| 4.1 | 5.2 | 7.8 | 15.8 | NTC | C1 | | 1.27 | 2.03 | 0.308271 | 1.933333 |
| 3 | 4.8 | 4.9 | 5.1 | PAPSS2 | C2 | | 1.60 | 1.04 | 0.007519 | -1.63333 |
| 5 | 4 | 10.4 | 13.5 | RPS6KB2 | C3 | | 0.80 | 1.30 | -0.59398 | 1.166667 |
| 4.7 | 3.9 | 10.1 | 15.1 | PFTK1 | C4 | | 0.83 | 1.50 | -0.66917 | 1.7 |
| 2.8 | 4.6 | 7.1 | 5.3 | NME5 | C5 | | 1.64 | 0.75 | -0.14286 | -1.56667 |
| 2.9 | 4.4 | 5.2 | 8.7 | SRC | C6 | | 1.52 | 1.67 | -0.29323 | -0.43333 |
| 4.1 | 4.9 | 8.4 | 9.4 | NEK4 | C7 | | 1.20 | 1.12 | 0.082707 | -0.2 |
| 6.1 | 3.9 | 11.4 | 10.7 | CSNK1G1 | C8 | | 0.64 | 0.94 | -0.66917 | 0.233333 |
| 3 | 4.4 | 5.7 | 9.2 | RIOK1 | C9 | | 1.47 | 1.61 | -0.29323 | -0.26667 |
| 3.4 | 1.9 | 6 | 3.1 | AKT1 | C10 | | 0.56 | 0.52 | -2.17293 | -2.3 |
| 1.5 | 5.2 | 2.9 | 9.4 | SNRK | C11 | | 3.47 | 3.24 | 0.308271 | -0.2 |
| 0.6 | 0.8 | 1.2 | 1.2 | CytoD | C12 | | 1.33 | 1.00 | -3 | -2.93333 |
| 2.6 | 5.2 | 4.5 | 12.6 | NTC | D1 | | 2.00 | 2.80 | 0.308271 | 0.866667 |
| 3.8 | 5.5 | 6.6 | 10.4 | KIAA1811 | D2 | | 1.45 | 1.58 | 0.533835 | 0.133333 |
| 4.6 | 5.5 | 8.2 | 9.4 | PDK2 | D3 | | 1.20 | 1.15 | 0.533835 | -0.2 |
| 1.7 | 3.5 | 3.8 | 7.1 | MYLK2 | D4 | | 2.06 | 1.87 | -0.96992 | -0.96667 |
| 5.9 | 6.3 | 11.1 | 14.3 | NEK11 | D5 | | 1.07 | 1.29 | 1.135338 | 1.433333 |
| 4.1 | 5.1 | 7.8 | 13.7 | KIAA1361 | D6 | | 1.24 | 1.76 | 0.233083 | 1.233333 |
| 2.8 | 5.3 | 5.5 | 8.9 | SNF1LK | D7 | | 1.89 | 1.62 | 0.383459 | -0.36667 |
| 4.1 | 5 | 7.9 | 11.7 | PTK9 | D8 | | 1.22 | 1.48 | 0.157895 | 0.566667 |
| 2.6 | 6.1 | 5.5 | 10.4 | LATS1 | D9 | | 2.35 | 1.89 | 0.984962 | 0.133333 |
| 3.6 | 6.5 | 6.3 | 9.8 | PFKP | D10 | | 1.81 | 1.56 | 1.285714 | -0.06667 |
| 2.2 | 5.9 | 4.3 | 8.7 | PRKG1 | D11 | | 2.68 | 2.02 | 0.834586 | -0.43333 |
| 2.1 | 1.1 | 3.4 | 1.8 | CytoD | D12 | | 0.52 | 0.53 | -2.77444 | -2.73333 |
| 2.8 | 4.8 | 6 | 11.2 | NTC | E1 | | 1.71 | 1.87 | 0.007519 | 0.4 |
| 3.5 | 5.5 | 6.4 | 12.2 | RIOK2 | E2 | | 1.57 | 1.91 | 0.533835 | 0.733333 |
| 2.2 | 5.5 | 4.6 | 8.3 | FRAP1 | E3 | | 2.50 | 1.80 | 0.533835 | -0.56667 |
| 6 | 5.1 | 11 | 13.4 | PDGFRL | E4 | | 0.85 | 1.22 | 0.233083 | 1.133333 |
| 2.9 | 6.3 | 5.6 | 13.6 | AK7 | E5 | | 2.17 | 2.43 | 1.135338 | 1.2 |
| 5 | 5.7 | 9.7 | 15 | EPHA10 | E6 | | 1.14 | 1.55 | 0.684211 | 1.666667 |
| 8.3 | 5.3 | 14 | 15.2 | STK17B | E7 | | 0.64 | 1.09 | 0.383459 | 1.733333 |
| 3.2 | 5.2 | 6.8 | 13.2 | NPR2 | E8 | | 1.63 | 1.94 | 0.308271 | 1.066667 |
| 3.8 | 6 | 9.6 | 15.2 | GOLGA5 | E9 | | 1.58 | 1.58 | 0.909774 | 1.733333 |
| 7.9 | 6.3 | 14.2 | 13.4 | STK24 | E10 | | 0.80 | 0.94 | 1.135338 | 1.133333 |
| 5.1 | 5.4 | 8.5 | 14.8 | DGKB | E11 | | 1.06 | 1.74 | 0.458647 | 1.6 |
| 4.3 | 2.7 | 2.5 | 2.3 | siR_Abl | E12 | | 0.63 | 0.92 | -1.57143 | -2.56667 |
| 1.9 | 4.8 | 5 | 8 | NTC | F1 | | 2.53 | 1.60 | 0.007519 | -0.66667 |
| 2.7 | 5 | 5 | 8.1 | STK25 | F2 | | 1.85 | 1.62 | 0.157895 | -0.63333 |
| 3.3 | 4.7 | 5.9 | 9.2 | CDC7 | F3 | | 1.42 | 1.56 | -0.06767 | -0.26667 |
| 2 | 3.2 | 4.4 | 5.1 | CAMK2G | F4 | | 1.60 | 1.16 | -1.19549 | -1.63333 |
| 7.9 | 4.9 | 13.3 | 13.3 | URKL1 | F5 | | 0.62 | 1.00 | 0.082707 | 1.1 |
| 5.9 | 6.3 | 11.3 | 13.8 | MAP3K9 | F6 | | 1.07 | 1.22 | 1.135338 | 1.266667 |
| 7 | 5.3 | 11.8 | 13.3 | GUCY2D | F7 | | 0.76 | 1.13 | 0.383459 | 1.1 |
| 3 | 1.5 | 6.3 | 3.1 | CAMKK2 | F8 | | 0.50 | 0.49 | -2.47368 | -2.3 |
| 6.3 | 5.9 | 11.4 | 10.1 | KIAA1639 | F9 | | 0.94 | 0.89 | 0.834586 | 0.033333 |
| 4 | 6 | 7.7 | 6.5 | CAMKIINALPHA | F10 | | 1.50 | 0.84 | 0.909774 | -1.16667 |
| 3.8 | 5.5 | 6.5 | 5.3 | KIT | F11 | | 1.45 | 0.82 | 0.533835 | -1.56667 |
| 2.1 | 0.2 | 4.2 | 0.7 | siR_Abl | F12 | | 0.10 | 0.17 | -3.45113 | -3.1 |
| 0.6 | 3.4 | 1.7 | 1.8 | NTC no BtGFP | G1 | | 5.67 | 1.06 | -1.04511 | -2.73333 |
| 1.7 | 4.7 | 3.8 | 9.1 | PRKCL1 | G2 | | 2.76 | 2.39 | -0.06767 | -0.3 |
| 3 | 5.1 | 5.9 | 12.1 | RIPK2 | G3 | | 1.70 | 2.05 | 0.233083 | 0.7 |
| 3.1 | 4.1 | 5.5 | 12.6 | CKMT1B | G4 | | 1.32 | 2.29 | -0.5188 | 0.866667 |
| 3.8 | 8.2 | 7 | 12.8 | MKNK2 | G5 | | 2.16 | 1.83 | 2.56391 | 0.933333 |
| 2.6 | 4.9 | 4.7 | 12.3 | TNNI3K | G6 | | 1.88 | 2.62 | 0.082707 | 0.766667 |
| 3.1 | 5.5 | 5.6 | 13 | ALS2CR7 | G7 | | 1.77 | 2.32 | 0.533835 | 1 |
| 1.8 | 4.8 | 3.5 | 8.7 | HCK | G8 | | 2.67 | 2.49 | 0.007519 | -0.43333 |
| 3.4 | 5.4 | 6.9 | 12.3 | CKS1B | G9 | | 1.59 | 1.78 | 0.458647 | 0.766667 |
| 4 | 4.1 | 7.5 | 12.6 | BMP2K | G10 | | 1.03 | 1.68 | -0.5188 | 0.866667 |
| 7 | 6.6 | 10.7 | 13.1 | PHKB | G11 | | 0.94 | 1.22 | 1.360902 | 1.033333 |
| 3.8 | 2.1 | 8.2 | 4.1 | siR_AKT1 | G12 | | 0.55 | 0.50 | -2.02256 | -1.96667 |
| 0.1 | 0.2 | 0.1 | 0.7 | NTC no BT_GFP | H1 | | 2.00 | 7.00 | -3.45113 | -3.1 |
| 3.5 | 5.4 | 5.7 | 4 | TRIO | H2 | | 1.54 | 0.70 | 0.458647 | -2 |
| 4.1 | 5.3 | 6.3 | 12.3 | ETNK1 | H3 | | 1.29 | 1.95 | 0.383459 | 0.766667 |
| 4.4 | 5.2 | 7.1 | 13.2 | ERK8 | H4 | | 1.18 | 1.86 | 0.308271 | 1.066667 |
| 4.2 | 3.4 | 7.9 | 5.3 | ADP-GK | H5 | | 0.81 | 0.67 | -1.04511 | -1.56667 |
| 6.6 | 3.5 | 10.5 | 3.3 | PIK3CG | H6 | | 0.53 | 0.31 | -0.96992 | -2.23333 |
| 3.5 | 6.1 | 5.9 | 12.2 | FGFR3 | H7 | | 1.74 | 2.07 | 0.984962 | 0.733333 |
| 4.9 | 6.8 | 8.4 | 10.1 | IRAK4 | H8 | | 1.39 | 1.20 | 1.511278 | 0.033333 |
| 4.6 | 7.4 | 7.6 | 12.9 | PCTK3 | H9 | | 1.61 | 1.70 | 1.962406 | 0.966667 |
| 3.3 | 6.2 | 5.8 | 13.7 | SGK2 | H10 | | 1.88 | 2.36 | 1.06015 | 1.233333 |
| 3.3 | 4.6 | 5.4 | 12.6 | DYRK4 | H11 | | 1.39 | 2.33 | -0.14286 | 0.866667 |
| 1.7 | 2.4 | 3.3 | 4.4 | siR_AKT1 | H12 | | 1.41 | 1.33 | -1.79699 | -1.86667 |

**Statistics**: A standard z-score was calculated based on the average values and standard deviations from the negative and positive control samples collected from all experimental plates: z = (x-µ)/σ, where x is % GFP-positive THP-1 cells from a single well, µ is the mean value calculated for the negative control samples (NTC) and σ is the standard deviation of all control samples versus the medium of the assay reads.

| Plate 8 | | | | |
| --- | --- | --- | --- | --- |
|  | First run | | Second run | |
|  | 4h | 24h | 4h | 24 h |
| average NTC, µ | 3.89 | 8.14 | 3.21 | 6.50 |
| average positive controls | 0.76 | 2.23 | 1.78 | 1.86 |

**Supplementary Table 2. Secondary validation screen with imaging flow cytometry.**

A differential two-step labeling method was applied to distinguish between intracellular and extracellular *B. thailandensis*. Extracellular *Burkholderia* adherent to the THP-1 membranes were double-labeled with FITC and PE fluorescence, whereas intracellular bacteria were single-labeled with FITC. The labeled THP-1 cells were analyzed using imaging flow cytometry (Amnis ImageStream^X^) with the following data collection parameters: slow flow rate, 60x magnification, and extended depth of field. Images were collected from the darkfield, brightfield, FL1 (488ex/560em nm), and FL2 (488ex/595em nm) channels. The IDEAS 5.0 software was used to determine the percentage of single versus double-labeled THP-1 cells.

|  | Frequencies of labeled THP-1 cells normalized to untreated | | | | | | | | | | | | | |
| --- | --- | --- | --- | --- | --- | --- | --- | --- | --- | --- | --- | --- | --- | --- |
|  | extracell | | intracell | | extracell | | intracell | | extracell | intracell | ave | | sdev | |
|  | FITC&PE | | FITC | | FITC&PE | | FITC | | FITC&PE | FITC | FITC&PE | FITC | FITC&PE | FITC |
| siR-CTL | 18 | | 68 | | 12 | | 38 | | 15 | 20 | 15.00 | 42.00 | 3.00 | 24.25 |
| siR-PTK7 | 3 | | 1.5 | | 6 | | 2 | | 8 | 5 | 5.67 | 2.83 | 2.52 | 1.89 |
| siR-CALM1 | 30 | | 25 | | 35 | | 18 | | 18 | 12 | 27.67 | 18.33 | 8.74 | 6.51 |
| siR-STK35 | 6 | | 1 | | 10 | | 1.8 | | 12 | 4 | 9.33 | 2.27 | 3.06 | 1.55 |
| siR-STK38L | 6 | | 2 | | 12 | | 4 | | 5 | 1 | 7.67 | 2.33 | 3.79 | 1.53 |
| siR-EPHB2 | 9 | | 3 | | 15 | | 5 | | 6 | 4 | 10.00 | 4.00 | 4.58 | 1.00 |
| siR-PKC | 3 | | 0.6 | | 8 | | 1.2 | | 9 | 4 | 6.67 | 1.93 | 3.21 | 1.81 |
| Ratio (extracellular/intracellular | | | | | | | |  |  |  |  |  |  |  |
|  |  |  |  |  |  |  |  |  |  |  |  |  |  |  |
| Exp1 | Exp2 | Exp3 | | ave | | stev | |  |  |  |  |  |  |  |
| 0.26 | 0.32 | 0.75 | | 0.44 | | 0.27 | |  |  |  |  |  |  |  |
| 2.00 | 3.00 | 1.60 | | 2.20 | | 0.72 | |  |  |  |  |  |  |  |
| 1.20 | 1.94 | 1.50 | | 1.55 | | 0.37 | |  |  |  |  |  |  |  |
| 6.00 | 5.56 | 3.00 | | 4.85 | | 1.62 | |  |  |  |  |  |  |  |
| 3.00 | 3.00 | 5.00 | | 3.67 | | 1.15 | |  |  |  |  |  |  |  |
| 3.00 | 3.00 | 1.50 | | 2.50 | | 0.87 | |  |  |  |  |  |  |  |
| 5.00 | 6.67 | 2.25 | | 4.64 | | 2.23 | |  |  |  |  |  |  |  |

**Supplementary Figure 1. Validation of RNAi screen using independent siRNAs to specifically reduce target gene expression.** To test the gene target specificity of the siRNA SMARTpool reagents, 1x10^6^ cells were treated with 50 nM siRNA and total RNA isolated 72 h post-treatment was applied in a One-Step RNA-to-Ct-value RT-PCR reaction (ThermoFisher Scientific) supplemented with Applied Biosystems™ TaqMan® Gene Expression Assays against the indicated gene transcripts. The RNA ratio for each target gene was determined relative to cells treated with non-targeting control (NTC) siRNA reagent and was normalized to the 18S ribosomal transcript for each reaction. RNA input was 40 ng per reaction. The statistics were obtained from three independent transfection experiments.


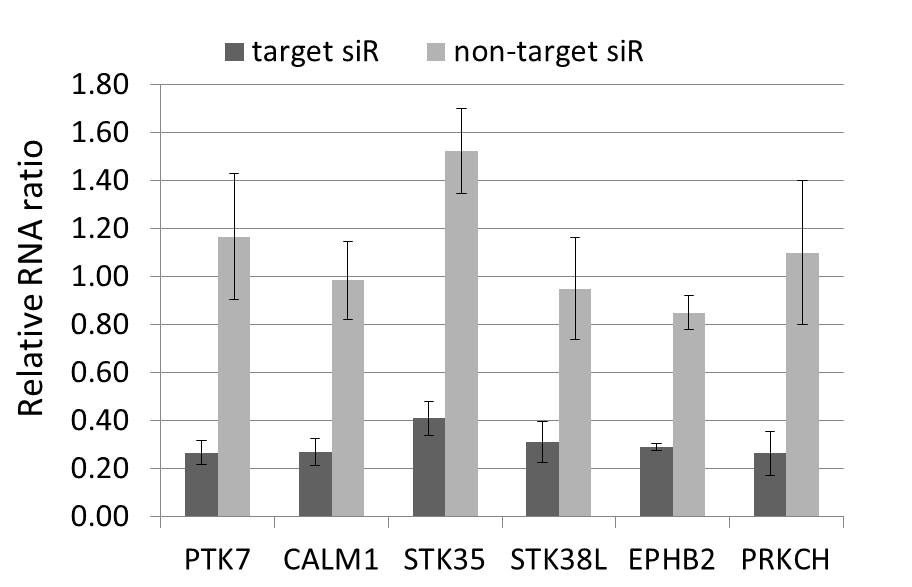


**Supplementary Figure 2. Downregulation of host genes by siRNA does not induce host cell death in bacteria free cells.** The cytotoxic effect of the siRNA SMARTpool reagents was evaluated using Applied Biosystems™ CellTiter-Glo® 2.0 Assay (Promega) to determine the number of viable host cells in culture by measuring the amount of ATP, which correlates to the amount of metabolically active cells. For each reaction we have counted the total cell count using trypan blue to exclude the amount of cells with compromised cell membranes.

**
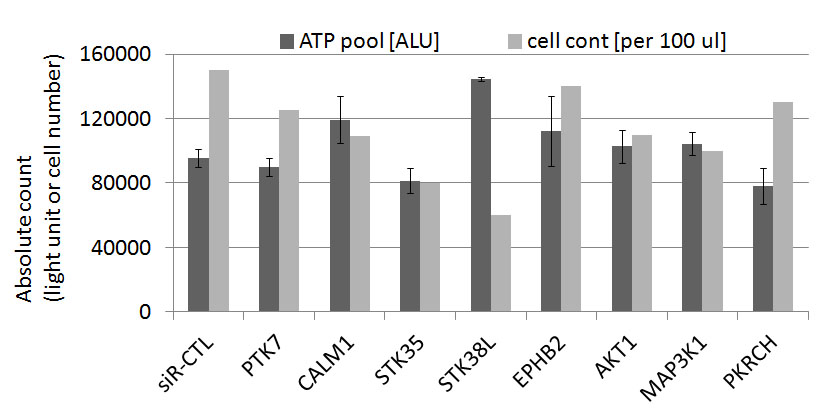
**

**Supplementary Figure 3. Western blot data on the specific reduction of PKC-η protein levels by siR_PRKCH.** Data was used to generate Figure 2B in the manuscript and demonstrates that the protein levels of classic PKC-α isoform remain unchanged in cells treated with siRNA targeting PKRCH transcripts (siR_PRKCH). THP-1 cells were treated with 50 nM siRNA targeting PRKCH transcripts or non-targeting control siRNAs. Total protein was isolated 72h post siRNA treatment using RIPA buffer (pH 8) to lyse the cells. 10 μg of protein from the pre-cleared whole cell lysates (WCL) were loaded on 4-10% gradient Mini-PROTEAN TGX Precast Gels (BIO-RAD) and resolved via gel electrophoresis. Equal amounts of protein from a single WCL sample were loaded independently to probe for PKCα, PKCη, and actin protein with sc-8393 (H-7), sc-215 (C-15), and sc-58673 (2Q1055) antibodies, respectively.

**
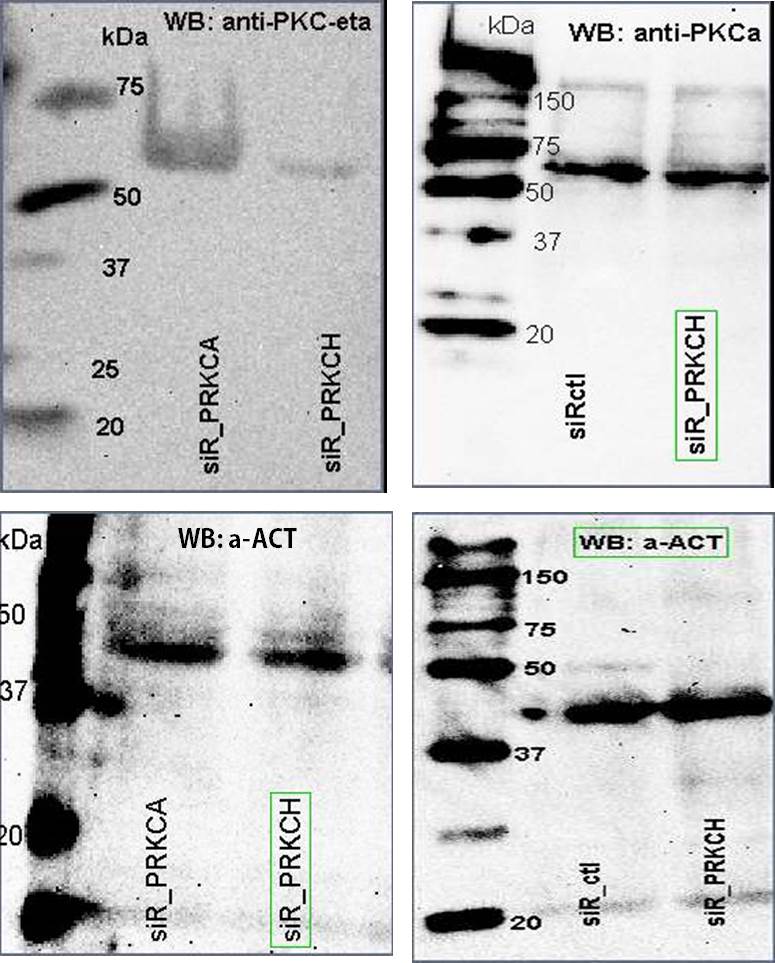
**
